# Syllable Complexity and Morphological Synthesis: A Well-Motivated Positive Complexity Correlation Across Subdomains

**DOI:** 10.3389/fpsyg.2021.638659

**Published:** 2021-03-17

**Authors:** Shelece Easterday, Matthew Stave, Marc Allassonnière-Tang, Frank Seifart

**Affiliations:** ^1^Department of Linguistics, University of Hawai'i Mãnoa, Honolulu, HI, United States; ^2^UMR 5596 Dynamique du Langage, Université Lumière Lyon 2 and Centre National de la Recherche Scientifique, Lyon, France; ^3^Leibniz-Centre General Linguistics (ZAS), Berlin, Germany

**Keywords:** complexity correlations, syllable structure, morphological synthesis, grammaticalization, language change, linguistic typology, corpus study

## Abstract

Relationships between phonological and morphological complexity have long been proposed in the linguistic literature, with empirical investigations often seeking complexity trade-offs. Positive complexity correlations tend not to be viewed in terms of motivations. We argue that positive complexity correlations can be diachronically well-motivated, emerging from crosslinguistically prevalent processes of language change. We examine the correlation between syllable complexity and morphological synthesis, hypothesizing that the process of grammaticalization motivates a positive relationship between the two features. To test this, we conduct a typological survey of 95 diverse languages and a corpus study of 21 languages with substantive (predominantly >10,000 words) corpora from the DoReCo project. The first study establishes a significant positive correlation between syllable complexity, measured in terms of maximal syllable patterns, and the index of synthesis (morpheme/word ratio). The second study tests the hypothesis that the relationship between syllable complexity and synthesis holds at local (word-initial and word-final) levels and within noun and verb types, as predicted by a grammaticalization account. While the findings of the corpus study are limited in their statistical power, the observed tendencies are consistent with our predictions. This study contributes important findings to the complexity literature, as well as a novel method which incorporates broad typological sampling and deep corpus analysis.

## Introduction

Studies of linguistic complexity are often undertaken with the aim of establishing trade-offs; that is, negative correlations between linguistic features. Empirical crosslinguistic studies in this vein typically seek to support or disconfirm the idea that all languages are of roughly equal complexity, a claim termed the “negative correlation hypothesis” (Shosted, 2006), the “trade-off hypothesis” (Sinnemäki, 2008), and the “hypothesis of equal complexity” (Nichols, 2009). Testing this axiom is problematic for a number of reasons, including definitional issues, the meaningfulness and appropriateness of the measures adopted, crosslinguistic comparability, the size and scope of the domains considered, whether the hypothesis is in fact falsifiable, and many other factors, some of which are explored by the contributions in this special issue. These complications aside, another problem is that the axiom itself does not explicitly state what motivates complexity trade-offs or the lack thereof. However, when established, negative complexity correlations are often interpreted as reflecting the self-organization of linguistic features in response to physiological and cognitive constraints (Fenk-Oczlon and Fenk, 2008; Oh et al., 2013; Coloma, 2016). Further, complexity trade-offs within the same domain are posited to be functionally motivated, reflecting efficiency in communication (Sinnemäki, this issue).

Non-correlations and positive correlations in complexity, on the other hand, are generally taken as evidence against complexity trade-offs, but are not typically discussed in terms of their own motivations, especially when they occur across domains. In a survey of the complexity of five grammatical domains in 130 languages, Nichols (2009) found no significant negative correlations between any of these domains, and a significant positive correlation between the complexity of synthesis and that of syntax, both of which were measured as composites of a collection of more specific features. Although two different correlational patterns were observed in the data – no correlation and a positive correlation – Nichols interpreted the general lack of negative correlations to be more meaningful, in that it ultimately yielded no support for the hypothesis of equal complexity. Similarly, in a sample of 32 languages, Shosted (2006) found a slightly positive but statistically insignificant correlation between the number of potential syllable types and the inflectional synthesis of the verb. He interpreted this as non-support for the negative correlation hypothesis, but otherwise did not take the slightly positive relationship to be meaningful in and of itself.

This paper seeks to address what we consider to be an intriguing but neglected question in the complexity literature: if trade-offs are considered to be motivated, synergetically, functionally, or otherwise, how do we interpret positive correlations in the complexity of linguistic features? Are they random, simply amounting to counterevidence for the complexity trade-off hypothesis, or can they, too, be well-motivated? And if they are motivated, then by which factors?

The paper is organized as follows. In section Background we present some background, discussing a case of a diachronically motivated positive complexity correlation within the domain of phonology, reviewing previous studies of correlations between phonological and morphological complexity, suggesting grammaticalization-related phonological reduction as a potential motivation for positive correlations between the subdomains, and introducing our research questions and hypotheses. We conduct two studies, one typological, and one corpus-based, the methodology of which is described in section Data and Methods. The results of these studies are presented in sections Typological Survey and Corpus Study, and we discuss their implications in section Discussion.

## Background

### A Well-Motivated Positive Complexity Correlation

One example of a potentially well-motivated positive complexity correlation is that of consonant phoneme inventory size and syllable structure complexity. In a sample of over 500 languages (Maddieson, 2006), established a weak but highly significant positive correlation between these two phonological features, a finding that has been confirmed in a number of subsequent studies using various measures of syllable complexity (Maddieson, 2011; Gordon, 2016; Easterday, 2019; Fenk-Oczlon, this issue). Because this trend holds when geographical region is controlled for, Maddieson suggests that the two features may be mutually reinforcing in their complexity, owing to “paths of natural historical linguistic change” (Maddieson, 2006: 118). Easterday (2019) further established that the presence of particular kinds of articulations in the consonant phoneme inventory, including richer place contrasts, is positively correlated with higher syllable complexity. In fact, there are a number of historically attested cases of vowel reduction phenomena which simultaneously increased syllable complexity and created new consonant contrasts, with coarticulatory remnants of the vowels being retained in the surrounding consonants. For example, in Lezgian, a process of pretonic high vowel syncope radically altered the syllable canon while adding a wide variety of consonants with contrastive secondary palatalization and labialization to the phoneme inventory (Haspelmath, 1993; Chitoran and Babaliyeva, 2007). A similar process has recently occurred in Nasa Yuwe: cf./βiˈtõ/: “stick” ca.1755 with its modern form, /ˈϕ^j^tũ/: (Díaz Montenegro, 2019: 178).

A synergetic approach might posit a negative correlation between consonant inventory size and syllable structure complexity, predicting that languages with fewer consonants would permit freer combinations of segments as a compensatory strategy [oman Jakobson, as reported by Saporta (1963); though see Fenk-Oczlon and Fenk (2008) for another interpretation]. Instead, we find a weak but consistent positive correlation between the two features, in line with the effects of observed processes of language change. Consonant inventory size and structure are theorized to be shaped by a wide range of factors, many of which are largely independent of syllable structure (Ohala, 1979; Lindblom and Maddieson, 1988; Stevens, 1989; Clements, 2003). Diachronic processes which introduce new phonemic contrasts may have no effect on canonical syllable structure, and syllable structure-affecting processes such as vowel epenthesis, cluster reduction, and vowel deletion do not necessarily impose changes upon the consonant inventory. However, we suggest that the subtle positive correlation is motivated at least in part by diachronic paths which affect and complexify both systems, like the historical processes mentioned above.

In this paper, we explore whether the forces of language change may similarly motivate positive complexity correlations between linguistic features from different subsystems of language, namely phonology and morphology.

### Correlations Between Phonological and Morphological Complexity

Proposed correlations between phonological and morphological complexity have been a central theme in holistic typologies for centuries (see Plank, 1998 for a review). Many such typologies predict an elaborate variety of specific phonological, morphological, syntactic, and semantic features which are expected to co-occur and are understood to be mutually supportive, both synchronically and diachronically. In some of these, properties of speech rhythm are hypothesized to drive the correlations (Donegan and Stampe, 1983; Gil, 1986; Auer, 1993). Syllable structure complexity, which bears a close relationship to speech rhythm (Ramus et al., 1999; Schiering, 2007; Easterday et al., 2011), features prominently among phonological features in most such typologies. Here we focus on empirical investigations seeking to establish correlations between syllable structure complexity and specific aspects of morphological complexity.

A series of studies by Gertraud Fenk-Oczlon and August Fenk have identified complexity trade-offs in this realm. In parallel sets of 22 unconnected simple declarative sentences from 26 predominantly Indo-European languages, Fenk and Fenk-Oczlon (1993) determined a significant negative correlation between the number of phonemes per syllable, a measure of syllable complexity, and the number of syllables per word, which they interpret to represent the complexity of the morphological subsystem. In similar data from a more diverse sample of 34 languages, Fenk-Oczlon and Fenk (2005) found a negative correlation between the same syllable complexity measure and the number of grammatical cases present in languages. A finer-grained study of eight Indo-European languages found a positive correlation between phonemes per syllable and the number of monosyllables in a language, but this was interpreted as a trade-off since higher numbers of monosyllables reflect low complexity in word structure (Fenk-Oczlon and Fenk, 2008).

Shosted (2006) tested the negative correlation hypothesis in a sample of 32 diverse languages. To measure phonological complexity, he calculated the potential number of distinct syllables from the number of phonemic contrasts, canonical syllable patterns, and reported phonotactic constraints for each language. The morphological complexity measure used was inflectional synthesis of the verb (Bickel and Nichols, 2005), which corresponds to the number of inflectional categories that can be simultaneously marked on the maximally inflected verb form. Shosted found a slightly positive but statistically insignificant correlation between the two measures. In a similar vein, Nichols (2009) reported that an earlier version of her study of complexity correlations in five linguistic domains found a significant positive correlation between phonology (a composite measure including consonant phoneme inventory size and syllable structure) and synthesis (a composite measure including inflectional synthesis of the verb, polyagreement, noun plural marking, and noun dual marking). This result was not replicated in the expanded published study of 130 languages.

Within a larger study of the properties of highly complex syllable structure, Easterday (2019) examined the correlation between syllable complexity and the index of synthesis in 63 diverse languages. Syllable complexity was measured in two ways, both defined according to consonant phonotactics: the first using a modification of the categorical typology in Maddieson (2006) which considers the size and shape of onset and coda patterns, and the second using the sum of the maximal onset and coda patterns measured in number of consonants. The index of synthesis is a quantitative measurement of morphological synthesis proposed by Greenberg (1954) and defined as the average number of morphemes per word in running text. It was found to have a positive correlation with syllable structure complexity when the latter was measured categorically (r(63) = 0.30, *p* < 0.05) and a slightly weaker positive correlation when it was measured as a sum of maximal syllable margins (r(63) = 0.26, *p* < 0.05).

There are a number of confounds in interpreting the results of the above studies. Each study compares syllable complexity with a different morphological feature or set of features. The theoretical motivations behind the choice of the features compared are not always clear, but may differ drastically. The studies differ in whether the feature values compared are typological (based on maximal or potential properties of the language as a whole) or corpus-based (reflective of average distributions within the system and in usage). When corpus measures are used, the size, naturalness, and comparability of the corpora differ. Similarly, the size and genealogical and areal diversity of the language samples range widely. The current work aims to address some of these confounds in the body of literature investigating correlations between phonological and morphological complexity.

### Grammaticalization: A Diachronic Source for A Positive Complexity Correlation?

Interestingly, three of the studies described in section Correlations Between Phonological and Morphological Complexity examine correlations between some measure of syllable structure complexity and some measure of morphological synthesis, yielding either non-correlations or positive correlations between the features. The motivations behind the choice of these two features in particular may seem obscure, as their functions in language are quite different. This very point has been remarked upon previously. Sinnemäki (2008) found that the functional load of different strategies for core argument marking – word order and head/dependent morphology – are inversely related to one another in a sample of 50 languages. He argues that these results support the idea that complexity trade-offs are more likely to occur between variables which serve related functions in language. Comparing his results to those of Shosted (2006), which found no trade-off between syllable complexity and morphological synthesis, he remarks that the diverging results of his study were due to the intentional choice of those functionally connected variables, “whereas the parameters studied by Shosted (2006) were functionally rather dissimilar” (Sinnemäki, 2008: 85).

We argue that while syllable complexity and morphological synthesis may be functionally dissimilar, occurring in entirely different subsystems of language, they are nonetheless similar in other important ways. Consonant phonotactics, a common measure of syllable complexity, concerns the grouping together of segments in syllable margins. Morphological synthesis concerns the grouping together of morphemes within a word. Due to this structural similarity, the two properties have the potential to coincide. In many languages, one or more of the consonants in a syllable margin may correspond exactly to an affixed or cliticized morpheme: e.g., Tzeltal /s-kuj-on/ 3a-believe-1abs “she believed me (to be a thief)” (Polian, 2013: 58); English *sixths* /siks-θ-s/ six-nmlz-pl. In such cases, syllable complexity and morphological synthesis are intertwined.

The patterns of phonetic vowel reduction and deletion which feed the complexification of syllable structure in a language are typically conditioned by stress (Easterday, 2019). The relationship of any resulting consonant clusters to morphological patterns can vary according to the environmental factors conditioning the process and other relevant properties of the language. For example, the process of pretonic vowel syncope in Lezgian mentioned above targets vowels in certain consonantal environments in the first syllable of the word, creating word-initial clusters. The resulting clusters are almost exclusively tautomorphemic and root-internal, since the language has very little, if any, productive prefixation (Haspelmath, 1993). By comparison, the syncope of metrically weak vowels in Mojeño Trinitario, a language with productive prefixation, has created a wide variety of tautomorphemic and heteromorphemic word-initial onset clusters (Rose, 2019). A full two-thirds of the 64 onset cluster types in a corpus of this language occur in heteromorphemic contexts, either exclusively or alongside tautomorphemic patterns for the same type (Rose, 2020).

The above cases show that phonologically conditioned vowel deletion may increase syllable structure complexity without any particular regard to the morphology, producing consonant clusters that overlap morpheme boundaries and clusters that do not. A different pattern is exhibited by Tzeltal, referenced above, in which tautosyllabic consonant clusters occur solely in the context of prefixation (Polian, 2013). This language does not have a strong stress system or any recent or ongoing processes of vowel reduction in the initial syllable^1^. Instead, the consonantal prefixes which initiate these complex onsets – h- (1a) s-/∫- (3a) and ∫- (incompl.i) – bear the hallmarks of highly grammaticalized elements. Grammaticalization is a process by which grammatical morphemes develop out of lexical morphemes. It involves the “dynamic coevolution of meaning and form,” with semantic reduction of the morpheme being accompanied by phonological reduction (Bybee et al., 1994: 20). Over the course of their development, grammatical morphemes may become very short and lose their autonomy, becoming strongly bound, phonetically and morphologically, to other elements and showing contextual allomorphy, like the Tzeltal prefixes. By the same token, already grammatical elements may continue along similar clines in what is known as “secondary” grammaticalization (Traugott, 2002).

It is important to note that, unlike the above scenario, grammaticalization may not involve phonological reduction at all, as phonetic erosion in this process depends on a variety of factors, including whether stress has segmental effects in a language (Schiering, 2010). Alternatively, grammaticalization can involve the phonetic and phonological reduction of grammatical markers without strong morphological fusion, as in Turkish “suffixes” (Zingler, 2018). In languages of East and Mainland Southeast Asia, many morphemes that denote grammatical functions exhibit neither phonological reduction nor morphological fusion (Bisang, 2004). In such scenarios, there is no reason to expect a direct overlapping of syllable complexity and morphological synthesis.

Given this variety of scenarios for both grammaticalization clines and the development of syllable structure complexity, we predict the following in terms of the interaction between syllable structure complexity and morphological synthesis. For languages with low syllable complexity, we expect that there will be a wide range of morphological synthesis values observed. For languages with higher degrees of syllable complexity, we expect a range of morphological synthesis values as well. In those languages we expect that many of the complex phonotactic patterns are the result of regular, phonetically conditioned processes of vowel reduction which operated without reference to the morphological environment. But we suggest that within this group, there are additionally languages in which phonological reduction associated with primary and secondary grammaticalization has produced consonantal affixes and clitics, leading to the emergence of consonant clusters in languages which otherwise do not have them, like Tzeltal.

Alternatively, such processes may expand the maximal syllable patterns in languages which already have clusters; for example, maximal codas in English, which occur only in the context of inflection: cf. *textes* ca. 1386 and modern *texts* /tεkst-s/. In either case, the grammaticalized consonantal morpheme is the locus of a direct overlapping of syllable complexity and morphological synthesis. We suggest that any positive crosslinguistic correlation between syllable complexity and morphological synthesis will be bolstered, at least in part and however subtly, by such cases. Thus, we are not proposing a universal relationship between syllable complexity and morphological synthesis, but a crosslinguistic tendency for high syllable complexity to cooccur with high values of morphological synthesis.

### The Current Study

The current study investigates the relationship between complexity in syllable structure and morphological synthesis: not because we take the two as proxies for phonological and morphological complexity, respectively, but because there is reason to believe that a relationship between these two particular features is theoretically well-motivated. In light of the discussion above, we hypothesize that processes of language change, and specifically grammaticalization, motivate a positive correlation between the two.

First, the current work aims to establish that there is a crosslinguistically robust association between syllable complexity and morphological synthesis. The sample used in Easterday (2019) consisted of 63 languages, but because the correlation effect found there was small (0.26–0.30), a larger sample size would improve the reliability of these results. Second, we test certain predictions of a grammaticalization account. All previous investigations into this topic have considered syllable complexity as a holistic value which is then compared against some similarly holistic measure of morphological synthesis. Yet a grammaticalization account also predicts local effects: that onset complexity will be correlated with morphological synthesis at the beginning of a phonological word, and that coda complexity will be correlated with morphological synthesis at the end of the word. This is exemplified by the Tzeltal and English examples mentioned above, in which maximal onset and coda patterns, respectively, are expanded by the presence of consonantal affixes. Further, a grammaticalization account would predict positive correlations between syllable complexity and morphological synthesis within parts of speech that tend to attract inflectional and other grammatical elements. Specifically, we would expect to find this positive relationship within both nouns and verbs, again as suggested by the English and Tzeltal examples.

Our research questions are: (1) Is there a positive correlation between syllable complexity and morphological synthesis, both broadly and on a local level? and (2) Is this correlation found within different parts of speech, specifically verbs and nouns?

We have designed two studies to address these questions, as well as some of the methodological issues of previous investigations mentioned in section Correlations Between Phonological and Morphological Complexity. Both compare measures of syllable complexity (in most cases defined according to consonant phonotactics) with some variation on the index of synthesis (morpheme/word ratio in running text, Greenberg, 1954). The first study is a broad survey of 95 languages in which various typological measures of syllable complexity are correlated with the index of synthesis derived from excerpts of narrative text. The second is a deeper study of naturalistic narrative corpora of 21 languages, in which we conduct a similar analysis and then analyze correlations between syllable complexity and indices of synthesis at the local (word-initial and word-final) level and at the level of word class (nouns and verbs). In the corpus study, we also test correlations between indices of synthesis and corpus-derived measures of syllable complexity. This study design allows us to explore complexity correlations in broad and deep ways, as well as to evaluate the comparability of typological and corpus-based measures within the same data set.

## Data and Methods

### Typological Survey

The sample used for the typological survey consists of 95 languages. This sample includes the 63 languages used for nearly identical analyses in Easterday (2019). In expanding the sample, languages with easily accessible morphologically annotated texts were selected from families that were un(der)represented in the previous sample. The current sample includes languages representing 82 top-level families, as classified in Glottolog (Hammarström et al., 2020), and 93 genera, as classified in the World Atlas of Language Structures (Dryer and Haspelmath, 2013). The sample languages are distributed over the six geographical macro-regions of the world (defined by Dryer, 1992: 84–85) as follows: Africa and Eurasia are represented by 11 languages each, Southeast Asia & Oceania by 13, Australia & New Guinea by 18, South America by 20, and North America by 22. Details of the sample can be found in Appendix A in Supplementary Material. The current sample reaches a statistical power of 0.847 when considering a medium effect correlation size (above 0.3). Because it is above the baseline of 0.8, the statistical power is considered sufficient for drawing conclusions from this data.

An additional design feature of both the previous and current samples is the deliberate representation of a wide variety of syllable patterns. In this sense, the sample displays typological bias (Comrie, 1989: 12), since patterns at the far ends of the syllable complexity cline are relatively overrepresented in the sample in comparison to their lower crosslinguistic frequencies. Using descriptions in reference materials, the syllable structure complexity of each language was coded in three ways, each defined by the consonant phonotactics of their canonical (maximal) syllable patterns:

**Categorical Syllable Complexity:** a four-level system in which languages are divided into Simple, Moderately Complex, Complex, and Highly Complex according to properties of their maximal onsets and codas. The categories are defined as follows. Simple: maximal onsets of one consonant and no codas; Moderately Complex: maximal onsets of two consonants, so long as the second is a liquid or glide, and maximal codas of up to one consonant; Highly Complex: maximal word-marginal sequences of three obstruents or four or more consonants; Complex: patterns which fall between Moderately Complex and Highly Complex (Maddieson, 2006, Easterday, 2019).

**Sum of Maximal Onset and Coda:** the sum of the number of consonants occurring in the maximal onset and coda of a language (Gordon, 2016, Easterday, 2019).

**Fine-Grained Sum:** same as above, but taking common sequencing profiles into account, much like the Moderately Complex category does for biconsonantal onsets in the Categorical Syllable Complexity classification. In this measure, if the closest consonant to the nucleus in the maximal onset is restricted to a liquid or glide, it counts as.5 rather than 1. Similarly, if the closest consonant to the nucleus in a maximal coda is restricted to a sonorant or a glottal consonant, it counts as.5 rather than 1. This measure is meant to represent a middle ground between the above two measures.

The 95 languages are roughly evenly distributed between the four levels of syllable complexity in the Categorical Syllable Complexity classification: the Simple category is represented by 25 languages, the Moderately Complex and Complex categories by 24 languages each, and the Highly Complex category by 22 languages. The Sum of the Maximal Onset and Coda ranges from 1 to 13 (median 3, mean 3.3). The Fine-Grained Sum ranges from 1 to 12 (median 2.5, mean 3.1). The syllable complexity values for each language can be found in Appendix A in Supplementary Material.

Morphologically annotated texts in reference materials were analyzed to determine the Index of Synthesis. The analyzed texts represent a variety of genres, but are nearly always third-person or first-person monological narratives. The Index of Synthesis was determined by hand counting the number of morphemes and the number of words in a section of text and dividing the former by the latter. The word and morpheme segmentations presented by the authors of the reference materials were taken at face value. Clitics were counted as corresponding to separate words if presented separately in the transcription, and as part of a larger word when presented as such. Similarly, reduplicants were counted as separate morphemes if segmented as such, but not if they were analyzed as part of the root in the annotation. Zero morphemes were excluded from morpheme counts. Hesitations and units with unknown segmentation (as indicated in the text) were also excluded.

On average, the section of text analyzed for each language was 299 words in length; This figure ranges from 69 words to 573 words, but for all but four languages clearly surpasses the 100-word length used in Greenberg's (1954) classic study. The Index of Synthesis ranges from 1.01 in Koho (Bahnaric, Austroasiatic) to 3.02 in Kalaallisut (Eskimo-Aleut), with the language sample showing a median of 1.70 and a mean of 1.78 for this value. This range is in line with the observations of morphological typology, in which Kalaallisut is often cited as a prototypical polysynthetic language, and Vietnamese, with an Index of Synthesis of 1, is often cited as a prototypical isolating language (Comrie, 1989). The word and morpheme counts for each language can be found in Appendix A in Supplementary Material.

### Corpus Study

The sample of languages studied here is a convenience sample of corpora from 21 languages, which nonetheless represents broad genealogical and areal diversity (see Appendix B in Supplementary Material). These corpora are currently being processed in the context of the DoReCo project (Paschen et al., 2020), with publication of the entire resource expected in 2022. The corpora were compiled during fieldwork in mostly small speech communities speaking mostly minority, and often endangered, languages. The data selected for inclusion in DoReCo project, and thus the current study, consist primarily of monological texts, most typically traditional narratives. We are not aware that potential slight genre differences within this data set (or in the textual data used in the typological study) would have an influence on the measures taken here, and therefore do not consider genre further in our analyses. Data were transcribed and morphologically analyzed and annotated for part-of-speech by experts on the respective languages. Corpus sizes range from 3,796 word tokens (Sanzhi Dargwa) to 52,111 (Pnar), with a median size of 15,884. Most analyses in this study are done from word types, which range from 1,343 (Savosavo) to 10,579 (Bora), with a median size of 3,404. In terms of statistical power, this sample of 21 languages reaches a power of 0.85 when considering a large effect size (correlation coefficient above 0.6). Any correlations with small or medium effect size that we find should thus be considered with a grain of salt.

In addition to genetic and areal diversity, the 21 languages were selected for having complete morphological annotation, including tiers for word, morph, and part of speech. Files were exported from ELAN to time-aligned, morpheme-level tabular format using the Multitool (Delafontaine, 2020), and were cleaned of extraneous characters and any words with incomplete or misaligned morphological information. Given the diverse nature of the transcription files, many languages required language-specific cleaning functions as well, for example, in order to exclude zero morphemes and pause markers.

First, we extracted morphological structure and word class information from the corpora. To isolate verbs and nouns, we relied on the expert part-of-speech annotations, which were mostly at the morpheme level, and rarely at the word-level. For files with word-level part-of-speech tags, these tags were used to identify word classes, and morpheme-separators were used to distinguish roots, prefixes, suffixes, infixes, proclitics, and enclitics.

For files with morpheme-level part-of-speech tags, to identify word classes, all part-of-speech tags in each corpus were associated with their corresponding word class (e.g., verb, noun, adverb, conjunction). Morpheme separators were used to distinguish affixes, clitics, and roots, and each word was then labeled with the part-of-speech of its root. Many languages, and particularly highly synthetic languages, had at least some words that were tagged as having multiple roots, as a result of processes like noun incorporation^2^. These were included in global measures such as index of synthesis or phonemes per syllable, but excluded from specific part-of-speech categorizations of their component roots. Words identified as borrowings were excluded from all analyses (between 0 and 7% of word types).

Next, we extracted syllable structure information. Because the corpora were not syllabified, we relied on word-initial and word-final consonant patterns to establish corpus-based distributions of onset and coda shapes. For each language, we converted the word and morph transcriptions to a SAMPA-based phoneme representation, utilizing grapheme-to-phoneme mappings used within the DoReCo project to perform phonemic time-alignment with the MAUS alignment software (Strunk et al., 2014). These mappings are created in collaboration with the corpus creators, and specify any graphemes that are not part of the language's orthographic system. Any words with such graphemes were considered to be borrowings, and were excluded from analysis. To establish onsets and codas, each phoneme was classified as a vowel or consonant, and we extracted word-initial and word-final consonantal patterns. Three languages (Goemai, Ruuli, and Sumi) exhibit syllabic consonants in certain contexts, and functions were written to correctly identify these.

From this data, we calculated a number of corpus-based measures of morphological synthesis. Apart from an initial analysis meant to stand in parallel to the broad typological survey, which uses Index of Synthesis as defined above, all subsequent analyses use corpus-based measures calculated over word types, rather than word tokens. The reasoning behind this is that the current study is not interested in frequency effects and highly frequent words could obscure language-wide patterns. The primary morphological measure is Index of Synthesis (Type), which is calculated as the mean number of morphemes per word type. We consider this measure within word classes: Index of Synthesis (Noun Type) and Index of Synthesis (Verb Type). Rather than take a particular stance on what constitutes a word, we have relied on the annotations of the language experts who created the corpora, which most often employ the phonological word, including proclitics and enclitics. We introduce local synthesis measures as well: the Index of Pre-Root Synthesis (Type) and the Index of Post-Root Synthesis (Type), which are the mean number of pre-root and post-root morphemes, including the root, per word type. These indices may also be specified for word classes (Noun, Verb).

Each language in the corpus sample was coded according to the typological measures of syllable complexity defined in section Typological Survey: Categorical Syllable Complexity, Sum of Maximal Onset and Coda, and Fine-Grained Sum. The individual components of the latter two measures – Maximal Onset and Maximal Coda, and the Fine-Grained versions of each – are also included in the analyses of local patterns here. Additionally, metrics meant to be corpus-based parallels to holistic syllable complexity and separate onset and coda complexity measures were calculated from the data. The mean number of Phonemes Per Syllable is taken as a corpus-based analog to the complexity of the whole syllable. Because the corpus is not syllabified, this is calculated as the ratio of phonemes to vowels and/or syllabic consonants within a word or word type. As analogs to Maximal Onset and Maximal Coda patterns, we take the mean length of word-initial and word-final consonant strings, also calculated over types: Avg. C Word-Initial (Type) and Avg. C Word-Final (Type). All corpus-based syllable complexity measures can also be specified for word class (Noun, Verb).

It is important to note that the languages in the corpus sample are quite skewed with respect to their syllable complexity measures: if we take the Categorical measure, there are 2 languages in the Simple category, 3 in the Moderately Complex category, 15 in the Complex category, and 1 in the Highly Complex category. The Sum of Maximal Onset and Coda ranges from 1 to 6 (median 3, mean 3.2), and the Fine-Grained Sum ranges from 1 to 5.5 (median 2.5, mean 2.9). Thus, there is a narrower range and less balanced dispersion of syllable patterns represented in this sample as compared to the typological sample. We admit that in addition to the small sample size, the limited diversity of syllable patterns in the corpus study may be a complicating factor in interpreting our results.

## Results

The quantitative analyses are conducted with the following R (R Core-Team, 2020) packages: brms (Bürkner, 2017), GGally (Schloerke et al., 2020), ggfortify (Tang et al., 2016), ggrepel (Slowikowski, 2019), lme4 (Bates et al., 2015), lmerTest (Kuznetsova et al., 2017), readxl (Wickham and Bryan, 2019), scales (Wickham and Seidel, 2020), sjPlot (Lüdecke, 2020), and tidyverse (Wickham, 2017).

### Typological Survey

**Figure 1:**
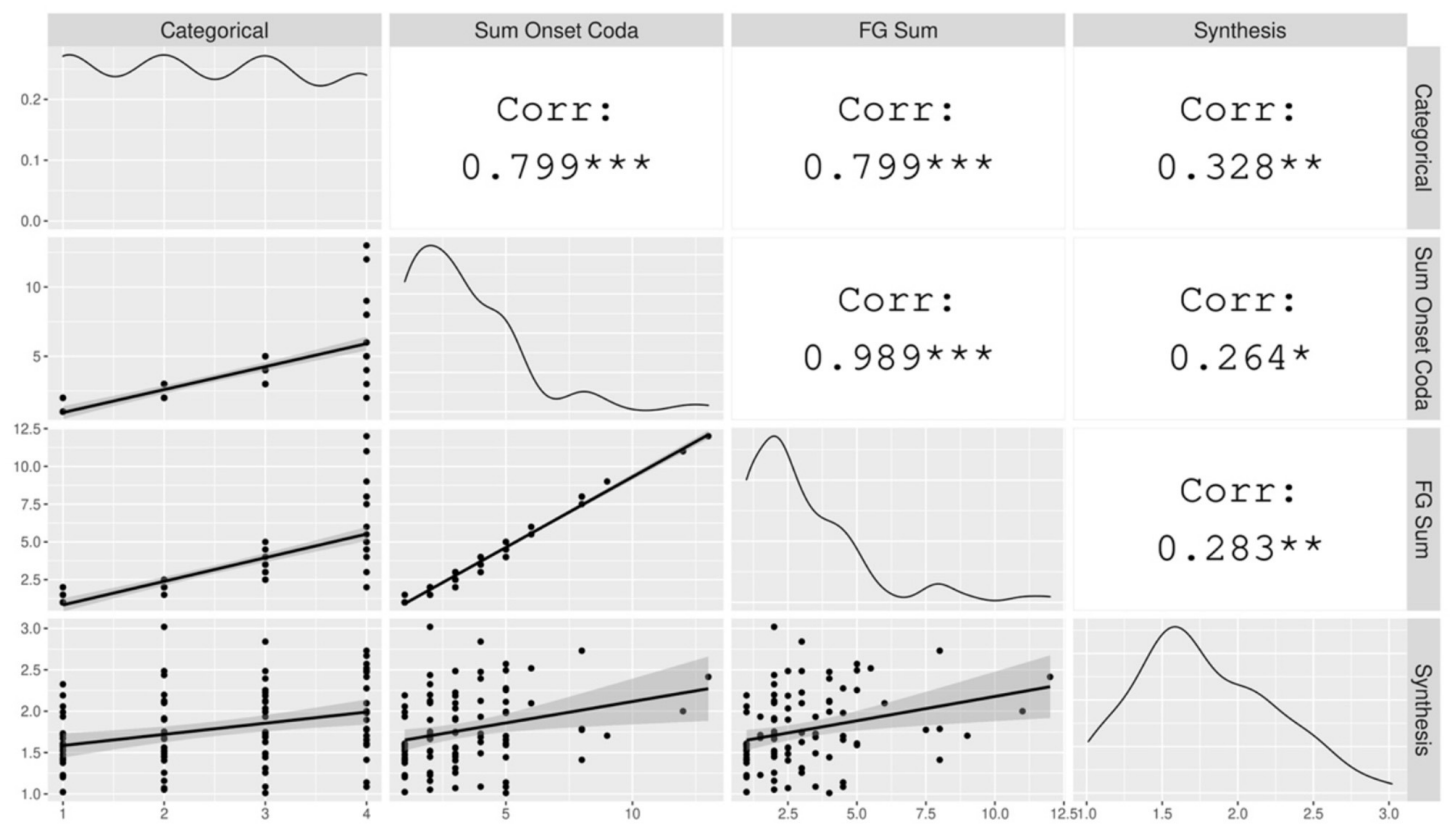
The correlation matrix of the variables included in the typological survey. The gray plots on the left hand show the data points with a linear regression line. The diagonal displays the distribution of each variable. The white cells on the right indicate the correlation coefficients and their statistical significance. The asterisks are interpreted as follows: *** = *p* < 0.001, ** = *p* < 0.01, * = *p* < 0.05, = *p* < 0.1, no asterisk = not statistically significant.

The hypothesis for the typological survey is that syllable structure complexity, measured according to the consonant phonotactics of the maximal syllable, and Index of Synthesis, derived from short narrative texts, are positively correlated. Correlation tests (Figure 1) show that the relationship is confirmed here for all three typological measures of syllable structure complexity: Categorical Syllable Complexity (r(95) = 0.33, *p* < 0.01), Sum of Maximal Onset and Coda (r(94) = 0.26, *p* < 0.05), and Fine-Grained Sum (r(94) = 0.28, *p* < 0.01)^3^. This range is similar to that found in the smaller sample of Easterday (2019). The observed correlations between syllable complexity and Index of Synthesis are significant but small^4^. We also observe that the different measures of syllable complexity are strongly correlated with each other (*r* ≥ 0.8), which confirms that they, as theoretically expected, convey similar information.

**Figure 2:**
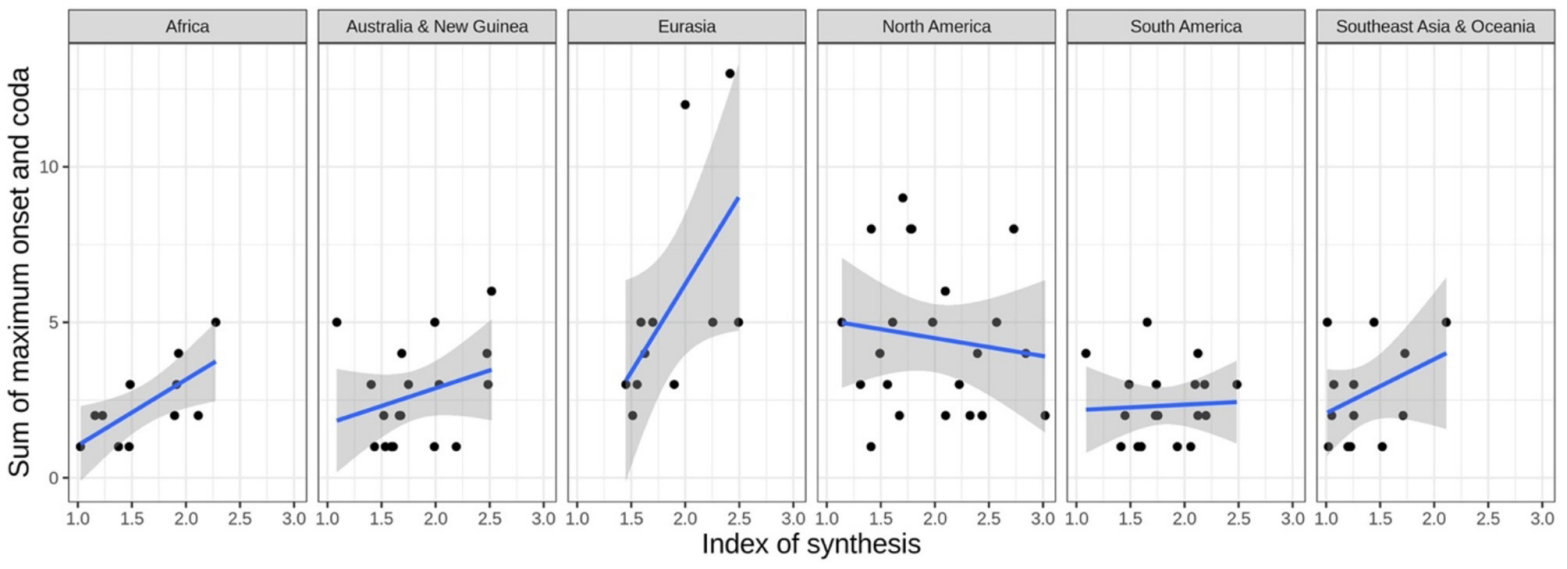
The relationship between Index of Synthesis and Sum of Maximum Onset and Coda across different geographical regions. Smoothed linear regression lines are shown in blue.

These correlation tests show the degree (correlation strength) and type of relationship (positive or negative) between pairs of variables. However, it is limited in the sense that, first, it does not say anything about how one variable affects another. Second, it does not take into account the variation that may occur across different geographical regions or genealogical groupings. Taking the interaction between Index of Synthesis and the Sum of Maximal Onset and Coda as an example, while we observe that they are weakly correlated, it is necessary to run regression-based tests to investigate how a change in the Sum of Maximal Onset and Coda affects the value of Index of Synthesis. With regard to the influence of area, while we observe a general correlation between Index of Synthesis and Sum of Maximal Onset and Coda in the entire data set, this correlation may vary across different areas and language families. As shown in Figure 2, the strength of the correlation varies across areas. For instance, the correlation is much stronger in Africa than in South America. Moreover, the relation between the two measures is negative in one area, North America, so this particular correlation would be a mere typological *trend* rather than a *preference* in terms of Dryer (1989). Similar effects are present across language families, which motivates the need to take into account the variation from genealogical and geographical effects.

To address these limitations, we test our hypotheses with linear mixed effects modeling. This modeling technique predicts the value of a dependent variable based on the predictor variable(s) while considering the effects of random grouping structures, which are specified as genera and areas in the current study to represent the random genealogical and geographical effects. Taking again the interaction between Index of Synthesis and the Sum of Maximal Onset and Coda as an example, the model uses the distribution of the Sum of Maximal Onset and Coda to predict the value of Index of Synthesis given the random structures of genus and area. For other examples of how this modeling technique is used in linguistic studies, please refer to Bentz and Winter (2013), Ladd et al. (2015), Sinnemäki and Di Garbo (2018), Sinnemäki (2019), and Sinnemäki (this issue).

Coefficients for the predictors' fixed effects are reported in Table 1. The output from three different models is reported. All three models consider genera and areas as random effects, while the predicted variable is the Index of Synthesis. Each of the three models uses one of the variables listed in Table 1 as a predictor, i.e., Categorical Syllable Complexity, Sum of Maximal Onset and Coda, and Fine-Grained Sum. We first observe that the coefficients (the estimates) are significant and positive for all three comparisons, which matches with the observations in our correlation-based analysis: the correlation between Index of Synthesis and each measure of syllable complexity remains positive^5^, even when controlling for genus and area. The coefficients are interpreted as follows. Taking once more the interaction between the Index of Synthesis and the Sum of Maximal Onset and Coda as an example, the coefficient is 0.046. This means that for an increase of one unit in the Sum of Maximal Onset and Coda, the Index of Synthesis increases by 0.046. This effect size is small and aligns with our previous tests showing that the correlation between the measures is present but weak. Nevertheless, the effect is not insignificant, since the range of the Sum of Maximal Onset and Coda is larger than the range of Index of Synthesis. For instance, an increase of 5 in the Sum of Maximal Onset and Coda would lead to an increase of around 0.23 for the Index of Synthesis, which represents a large leap since the average range of Index of Synthesis is about 1.0.

**Table 1:** Coefficients for the models with Index of Synthesis as the predicted variable and different measures of syllable complexity as predictors.

	**Parameters**	**Estimate**	**Std.error**	***t*-value**	***p*-value**
Synthesis ~ categorical	(Intercept)	1.544	0.111	13.907	0.000
	Categorical	0.087	0.032	2.706	0.033
Synthesis ~ sum max onset and coda	(Intercept)	1.595	0.085	18.763	0.000
	Sum Max Onset and Coda	0.046	0.011	4.387	0.029
Synthesis ~ fine-grained sum	(Intercept)	1.588	0.088	18.082	0.000
	Fine-Grained Sum	0.053	0.015	3.545	0.014

**Figure 3:**
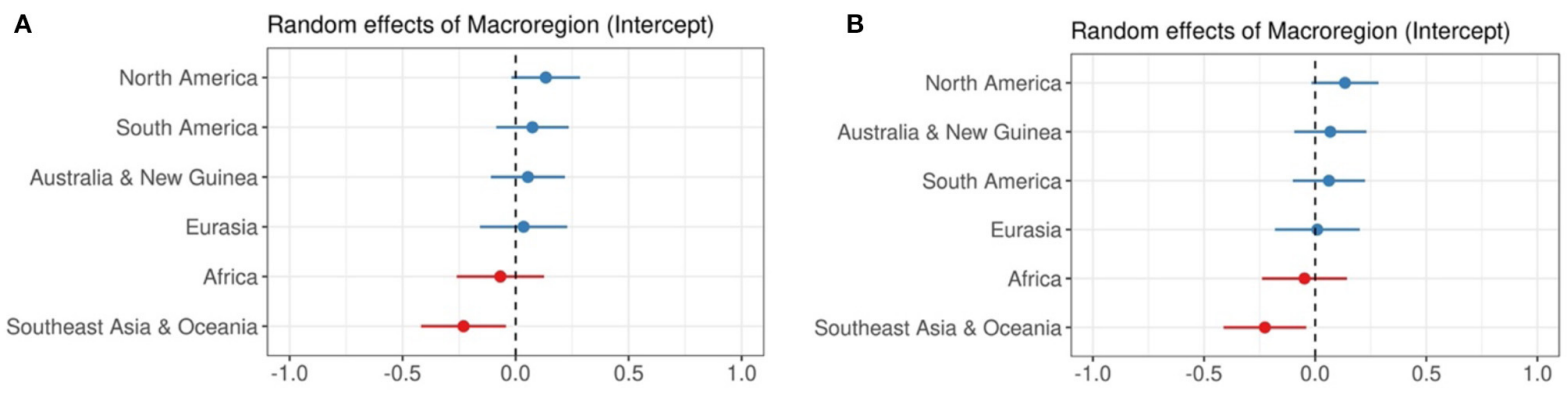
Random effects of macroregion when predicting Index of Synthesis. **(A)** Predictor: Categorical Syllable Complexity, **(B)** Predictor: Sum of Maximal Onset and Coda.

We also consider the random effects in the data. When considering areal effects, we observe that most areas do not have a significant areal effect on Index of Synthesis, except for Southeast Asia & Oceania, which as a region tends to have a lower Index of Synthesis. This effect, which is clear in Figure 3, is unsurprising, given the high concentration of isolating languages in the Southeast Asia portion of the region.

We do not display the full list of genealogical effects here. Since there is almost a one-to-one correspondence between languages and genera, there are no major biases in the data introduced by genus. Genera corresponding to extrema in the sample include Eskimo (Kalaallisut, mentioned above), Paya (Pech), and Caddoan (Wichita), which have the highest values for Index of Synthesis, and Bahnaric (Koho, mentioned above), Burmese-Lolo (Nuosu Yi), and Eastern Mande (Mann), which have the lowest values. Additional details on the output of the models are available in the Supplementary Materials.

### Corpus Study

#### Index of Synthesis and Syllable Complexity

First we consider the interaction between Index of Synthesis and different typological and corpus-based measures of syllable complexity in an analysis which is parallel to the one just presented for the typological survey. Following our hypothesis, we expect that the positive correlation will hold within the corpus sample.

**Figure 4:**
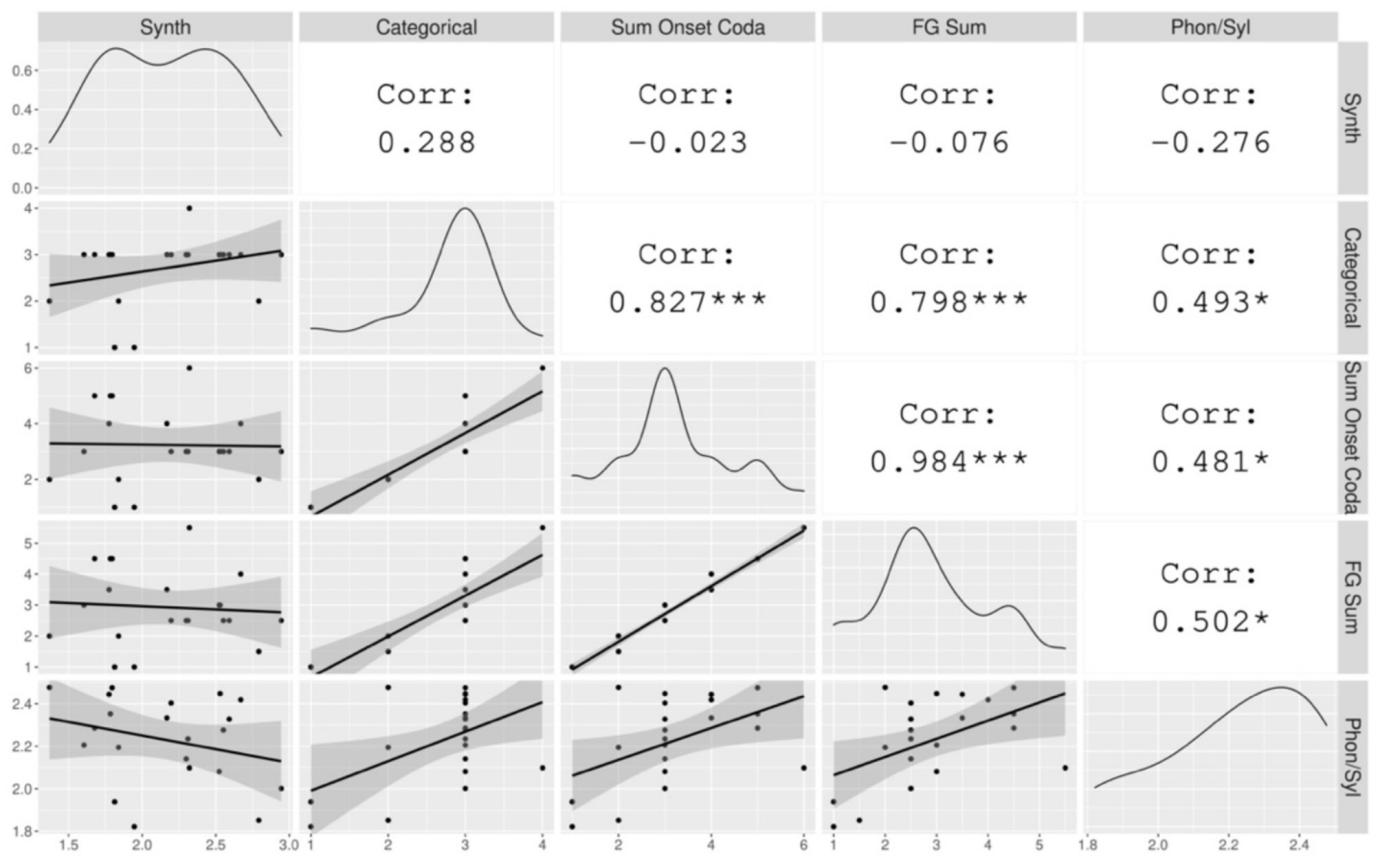
The correlation between Index of Synthesis and measures of syllable complexity. The gray plots on the left hand show the data points with a linear regression line. The diagonal displays the distribution of each variable. The white cells on the right indicate the correlation coefficients and their statistical significance. The asterisks are interpreted as follows: *** = *p* < 0.001, ** = *p* < 0.01, * = *p* < 0.05, = *p* < 0.1, no asterisk = not statistically significant.

Results are shown in Figure 4, in which the Index of Synthesis (calculated over tokens for this analysis) is plotted against typological measures of Categorical Syllable Complexity, Sum of Maximal Onset and Coda, and Fine-Grained Sum, and also against the corpus-based measure Phonemes Per Syllable (also calculated over tokens for this analysis). Within this sample, we do not find a significant correlation between any measure of syllable complexity and Index of Synthesis. This is to be expected, as the positive correlation observed in the typological survey was small (around 0.30) and the analysis here is limited by a much smaller sample size. While we do not observe significant correlations between the two features here, we do visualize a weak positive relationship between Categorical Syllable Complexity and Index of Synthesis (r = 0.205, n.s.), the pair that showed the strongest correlation in the typological survey.

On the other hand, we find strongly positive significant correlations between the various typological measures of syllable complexity, as also observed in the typological survey. We can also visualize weaker correlations between each of these and the corpus-based metric, Phonemes Per Syllable. The weaker correlations among the latter pairs are unsurprising, given that the typological measures reflect the maximal potential of the language and the corpus-based measure reflects an average over language-specific frequency distributions of syllable types. Due to the small sample size, we do not consider the use of mixed models here, as there is a one-to-one correspondence between languages and genera.

#### Index of Synthesis in Parts of Speech and Syllable Complexity

**Figure 5:**
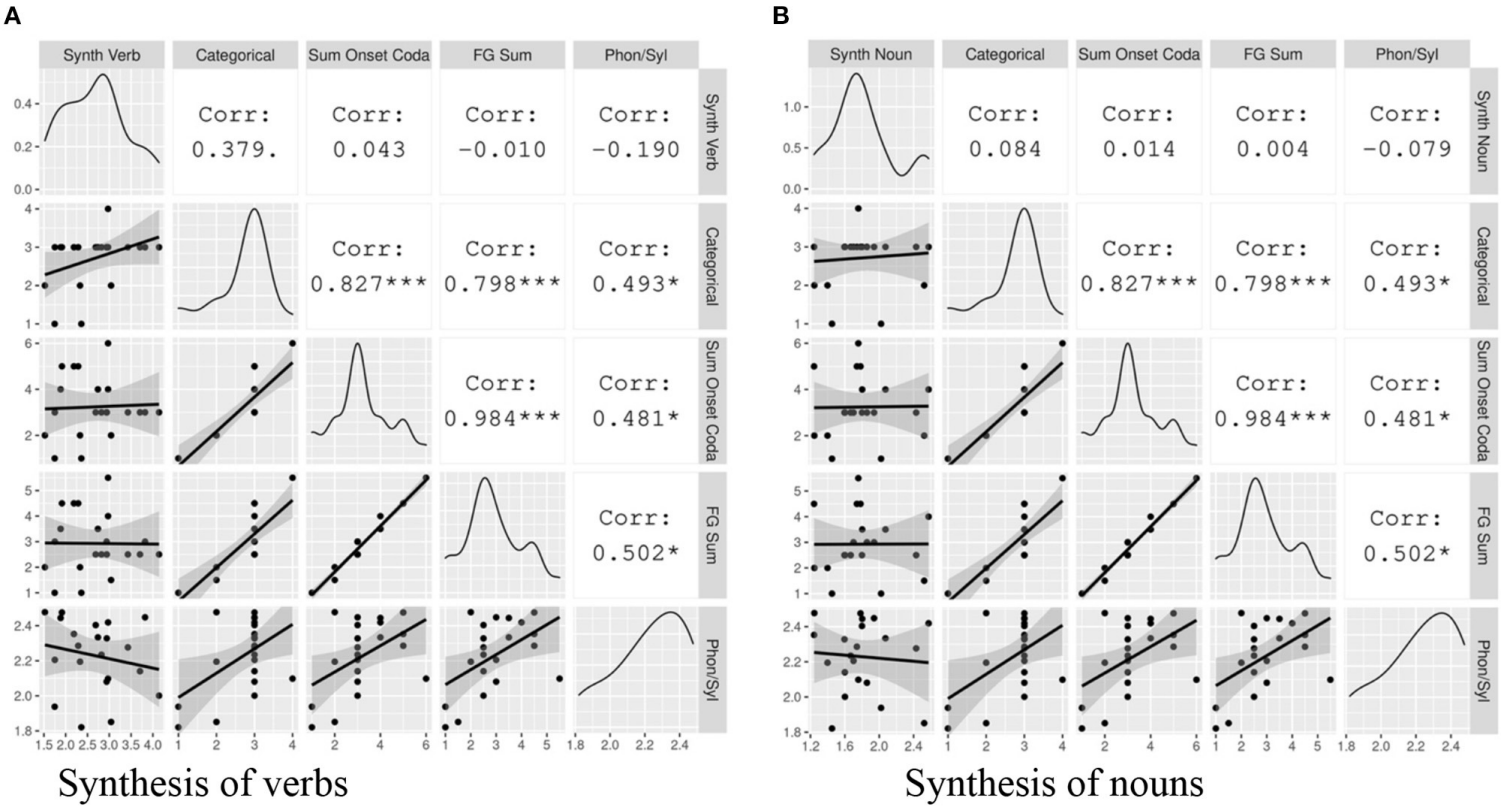
The correlation between Index of Synthesis (Verb Type) and Index of Synthesis (Noun Type) and measures of syllable complexity. The gray plots on the left hand show the data points with a linear regression line. The diagonal displays the distribution of each variable. The white cells on the right indicate the correlation coefficients and their statistical significance. The asterisks are interpreted as follows: *** = *p* < 0.001, ** = *p* < 0.01, * = *p* < 0.05, = *p* < 0.1, no asterisk = not statistically significant.

Before conducting an analysis of local patterns in the data, we address the second research question, which seeks to determine whether the correlation between syllable complexity and morphological synthesis can be found within different parts of speech, namely nouns and verbs. In addition to using typological syllable complexity measures, here we use Index of Synthesis and Phonemes Per Syllable metrics calculated over word types in the relevant part of speech. Figure 5 shows the correlations between the various measures of syllable complexity and the Index of Synthesis (Verb Type) (Figure 5A) and Index of Synthesis (Noun Type) (Figure 5B).

None of the correlations between syllable complexity and Index of Synthesis in these plots are statistically significant below the level of *p* < 0.05. Visualizing the general tendencies in the plots with linear regression lines, we see that the Index of Synthesis (Verb Type) has a much steeper slope when plotted against Categorical Syllable Complexity in comparison with Index of Synthesis (Noun Type); indeed, this effect is significant at the level of *p* < 0.1. We take this to suggest that it is the synthesis of the verb that more strongly drives the correlation between syllable complexity and morphological synthesis. However, due to the small correlation and the small sample size, it is not possible to quantitatively verify that with the data at hand. Interestingly, the Phonemes Per Syllable metric shows a weak negative relation with the Index of Synthesis for both parts of speech.

**Figure 6:**
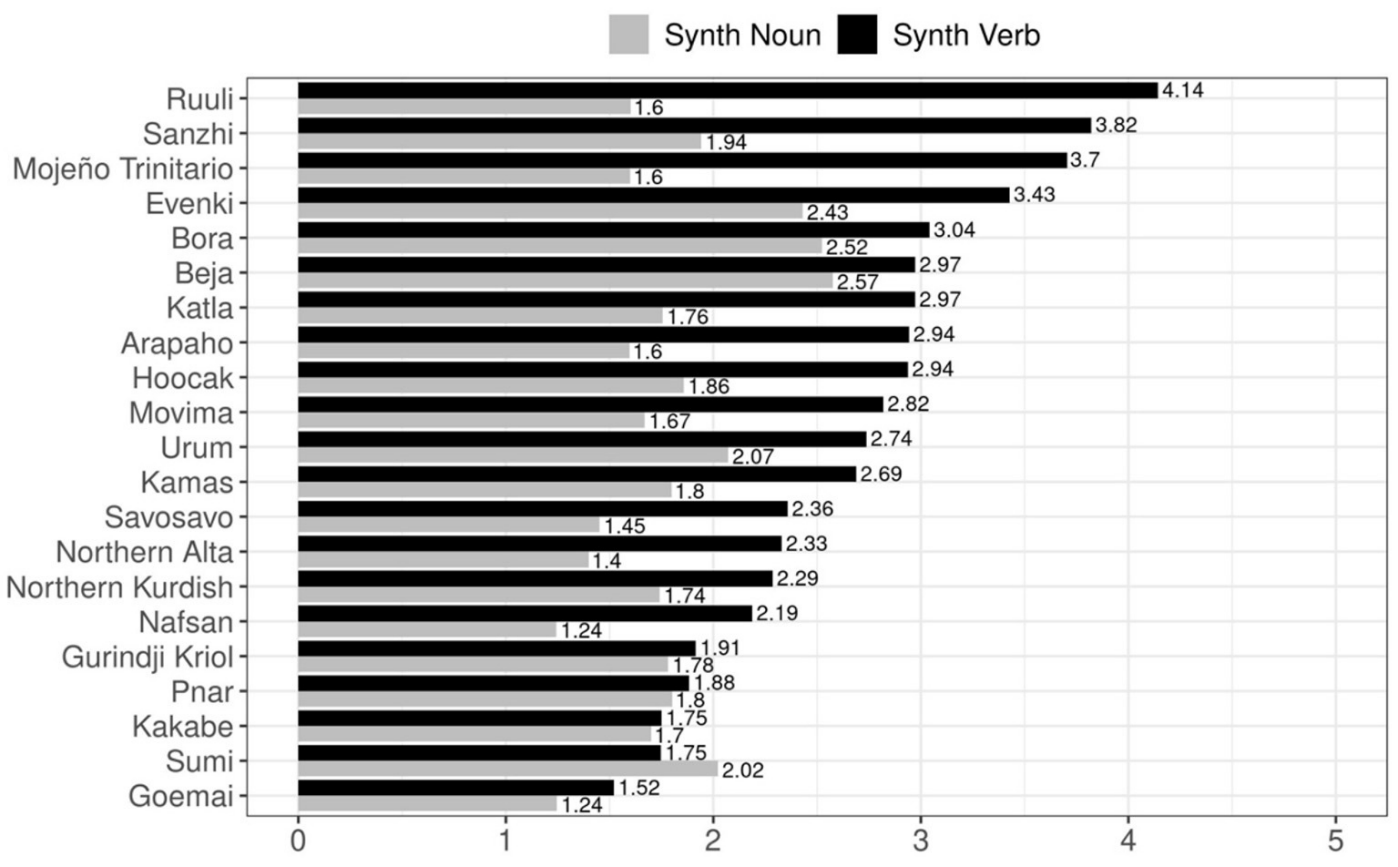
The index of synthesis for verbs and nouns across the languages of the corpus study.

A visualization of the indices of synthesis per language is provided in Figure 6. We observe that, as found in the correlation plots, the overwhelming trend is for the Index of Synthesis (Verb Type) to be higher than the Index of Synthesis (Noun Type) within languages. Only Sumi has a higher Index of Synthesis for noun types. Its relatively high value for this index is still much lower than most of the verbal synthesis measures for the other languages of the sample. There are also several languages which have very similar values for the two indices: Gurindji Kriol, Kakabe, and Pnar. Notably, these languages all have smaller than average values for Index of Synthesis in verb types (average = 2.68 morphemes/verb).

As there is much less crosslinguistic variation in the Index of Synthesis (Noun Type) values than in the verbal equivalent (a range of 1.24–2.57 for the former vs. 1.52–4.14 for the latter), it is unsurprising that any positive relationship this metric bears to syllable complexity in the current sample is small and non-significant.

#### Local Measures of Synthesis and Syllable Complexity

As stated above, a grammaticalization account predicts that the correlation between syllable structure complexity and morphological synthesis should occur not only globally but also at the local level; namely, in word-initial and word-final contexts. Here we test that hypothesis, examining the relationship between local indices of synthesis and local typological and corpus-based measures of syllable complexity.

##### Pre-root Synthesis

**Figure 7:**
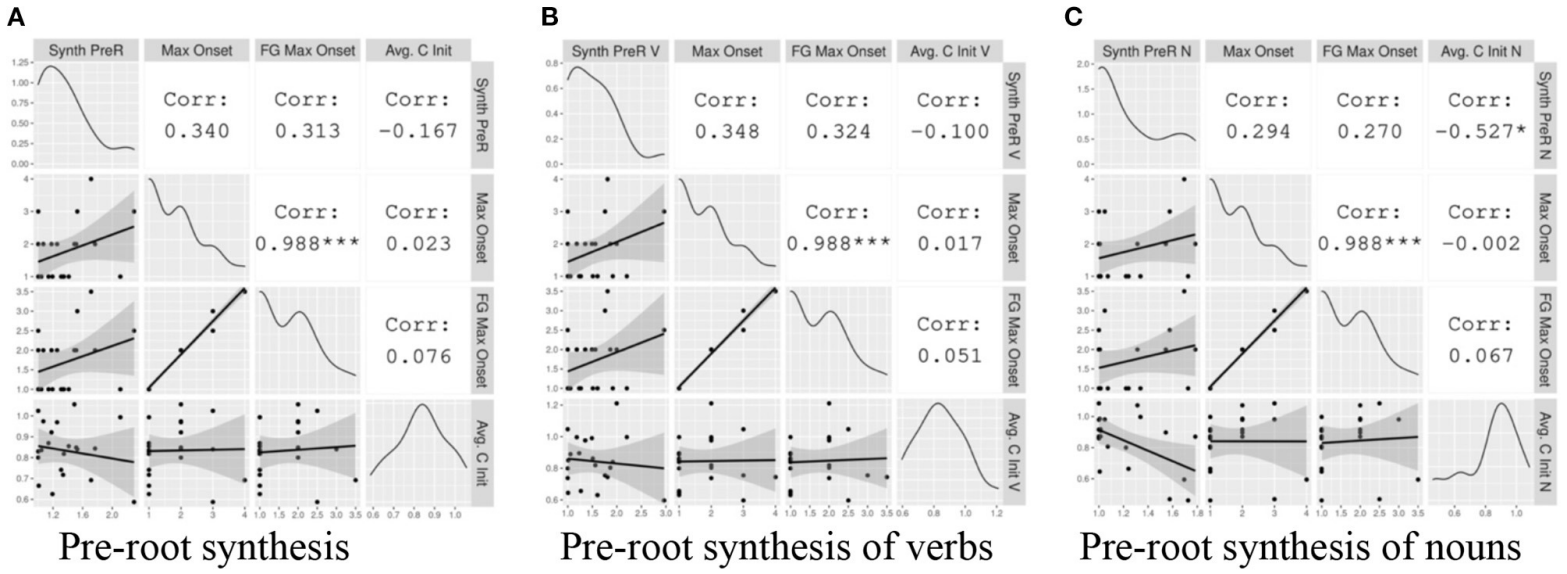
The correlation between local pre-root indices of synthesis and measures of onset complexity. The gray plots on the left hand show the data points with a linear regression line. The diagonal displays the distribution of each variable. The white cells on the right indicate the correlation coefficients and their statistical significance. The asterisks are interpreted as follows: *** = *p* < 0.001, ** = *p* < 0.01, * = *p* < 0.05, = *p* < 0.1, no asterisk = not statistically significant.

Here we examine word-initial local patterns. In Figure 7A we present the correlations between the Index of Pre-Root Synthesis (Type) and three syllable complexity measures: Maximal Onset and Fine-Grained Maximal Onset, both typological measures; and Avg. C Word-Initial (Type), a corpus-based measure. Figures 7B,C show the same correlations, but with the corpus-based synthesis and syllable complexity measures specified for Verb Type and Noun Type, respectively.

Within this small sample, most of the correlations between onset complexity and pre-root synthesis observed in Figure 7 do not reach statistical significance. However, examining the general tendencies, we visualize weak positive relationships between the typological measures of onset complexity and Index of Pre-Root Synthesis for word types in general, as well as within Verb Types and Noun Types. This is consistent with the predictions of the hypothesis.

On the other hand, we observe a weaker negative relationship between the corpus-based measure of onset complexity, Avg. C Word-Initial (Type), and the general and Verb Type indices of pre-root synthesis. However, the negative correlation between this metric and the Index of Pre-Root Synthesis (Noun Type) is both moderate and statistically significant (r = −0.53, *p* < 0.05). We will return to this point in section Discussion.

##### Post-root Synthesis

**Figure 8:**
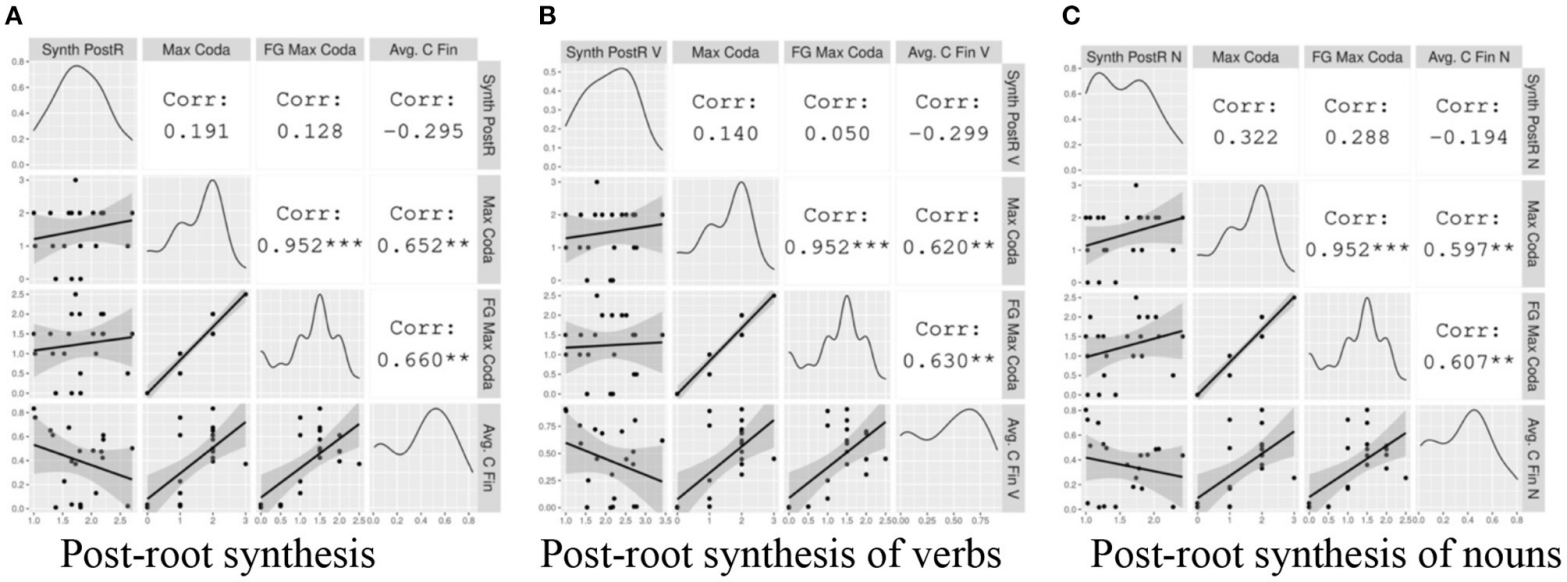
The correlation between local post-root indices synthesis and measures of coda complexity. The gray plots on the left hand show the data points with a linear regression line. The diagonal displays the distribution of each variable. The white cells on the right indicate the correlation coefficients and their statistical significance. The asterisks are interpreted as follows: *** = *p* < 0.001, ** = *p* < 0.01, * = *p* < 0.05, = *p* < 0.1, no asterisk = not statistically significant.

Here we present a similar analysis as in section Pre-Root Synthesis, focusing on local patterns in the word-final context. In Figure 8A, we show correlations between the Index of Post-Root Synthesis (Type) and Maximal Coda, Fine-Grained Maximal Coda, and Avg. C Word-Final (Type). Figures 8B,C show the same correlations, but with the corpus-based synthesis and syllable complexity measures specified for Verb Type and Noun Type, respectively.

Within this small sample, none of the correlations between coda complexity and Post-Root Synthesis reach statistical significance. Examining the general tendencies, we again visualize positive relationships, albeit generally weaker than for the word-initial context, between the typological measures of coda complexity and all Indices of Post-Root Synthesis. This is consistent with the predictions of the hypothesis.

We again visualize weak negative relationships between the corpus-based coda complexity metric, Avg. C Word-Final (Type), and Indices of Post-Root Synthesis for all word types. In some cases this relationship is stronger than the positive trend obtained from the typological measures.

#### Summary of the Interaction Between the Measures

Finally, we can use Principal Component Analysis (PCA) to investigate how the languages in the datasets can be differentiated based on the encoded variables. Principal component analysis is a technique used for unsupervised dimension reduction (Jolliffe, 2002). High dimensional data often include variables that are correlated and/or carry similar information. If the dataset is large, it is preferable to reduce it first before feeding it to other downstream tasks, hence the need for reducing the dimensions of the data. PCA fulfills this aim by using a mathematical procedure to transform a number of correlated variables into uncorrelated variables, which are called principal components. The first component accounts for as much of the variance in the data as possible. The embedded variance then decreases gradually in each of the following components. If only two components can explain most of the variance, the data size is substantially reduced, which is then very helpful for further processing. This method is widely used in areas such as image processing, genomic analysis, and information retrieval, among others.

**Figure 9:**
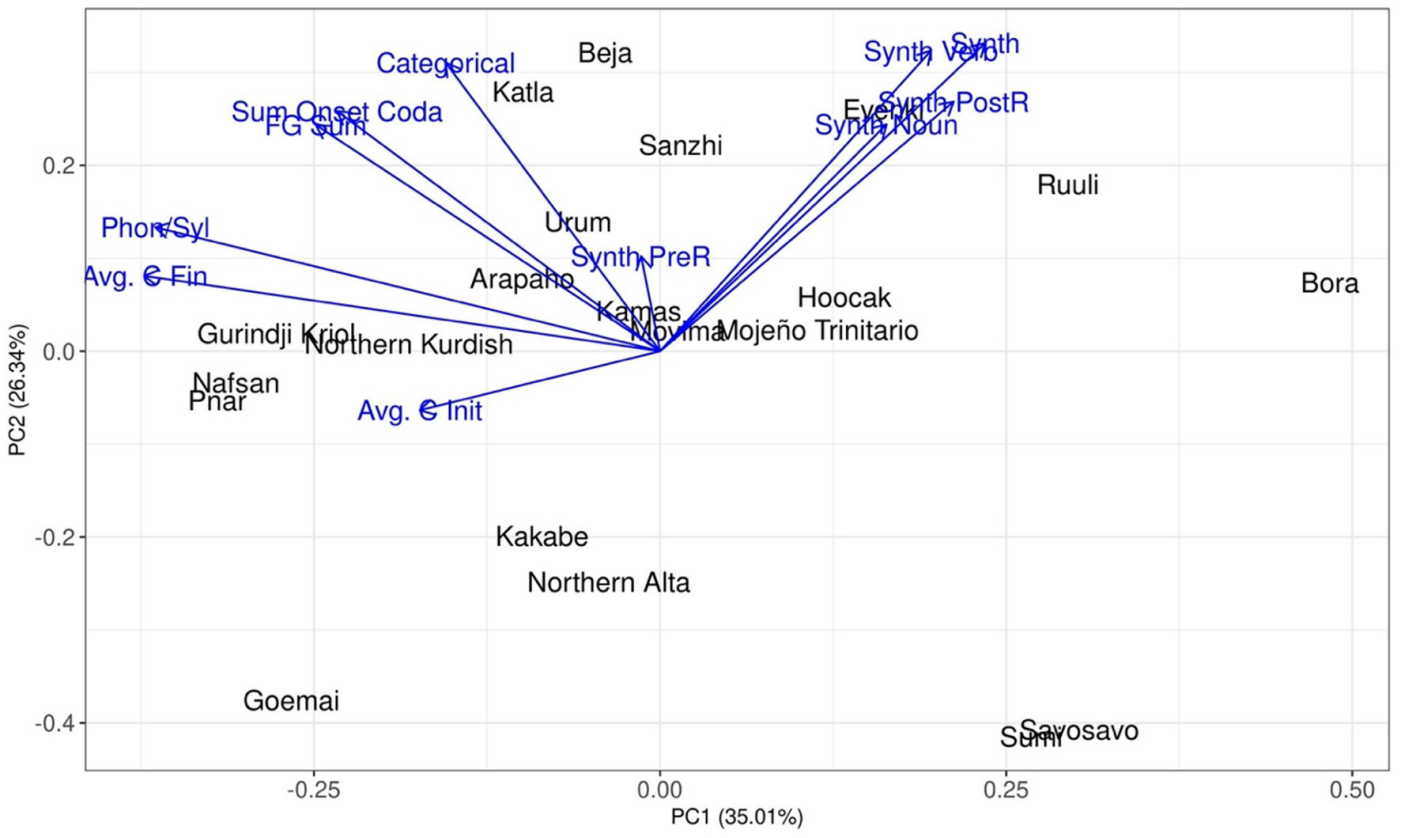
The interaction of the measures visualized by principal component analysis.

The PCA visualization of all the variables included in the corpus data is shown in Figure 9. Each point represents a language in the dataset. The distance between the languages reflects the similarities and dissimilarities across the encoded variables (e.g., different indices of synthesis, different measures of syllable complexity). The more similar two languages are based on the variables, the closer they are in the two-dimensional space. The arrows indicate the influence of each variable. The longer the arrow, the larger its influence. The direction of the arrows can also identify the specialties of the languages. For instance, Ruuli has generally high indices of synthesis, so it is found near the extreme ends of the arrows of the variations on this measure. A variable with a short arrow infers that the variable has similar values across all the languages of the data set.

Two general tendencies are found. First, most measures of Index of Synthesis point in the same direction. This matches with the correlation analyses performed in section Corpus Study: most of the different measures of Index of Synthesis behave in a similar way. Only one exception is found: the Index of Pre-Root Synthesis. Likewise, most measures of syllable complexity point in the same direction, which also shows that most of these measures convey similar information. However, the level of overlap of syllable complexity measures is not as strong as the measures of Index of Synthesis (as the arrows are more spread out than for measures of Index of Synthesis). This means that while different measures of Index of Synthesis convey almost identical information, different measures of syllable complexity convey similar, but not identical, information.

## Discussion

The results of our typological survey show that there is a crosslinguistically robust positive correlation between syllable complexity, measured according to the consonant phonotactics of maximal syllable structures in a language, and morphological synthesis, measured according to the Index of Synthesis. This correlation is small (r = 0.26–0.33) but statistically significant, and holds up within most geographic regions in a genealogically diverse sample. While the corpus study is too small to yield significant results for moderate and small correlations, the data suggests that a positive relationship is upheld there as well, particularly when Index of Synthesis is correlated with Categorical Syllable Complexity, the most coarse-grained of the measures. That correlation was the strongest of those established in the typological survey.

Moreover, the corpus-based study yielded support for the hypothesis, derived from a grammaticalization account, that the positive correlation between syllable complexity and morphological synthesis should be observable at the local level and within inflection-heavy parts of speech (verbs and nouns). Although the data suggests that it is the verb that more strongly drives the global correlation, we find local effects in the expected direction for verbs, nouns, and word types in general. This effect seems to be slightly stronger in word-initial contexts than in word-final contexts.

We find very different, and usually negative, correlations when the various indices of synthesis are related to corpus-based measures of syllable complexity. We suggest that this is because corpus-based measures capture the mean, which reflects frequency distributions of syllable patterns in a language. It is well-known that CV and CVC syllable types overwhelmingly predominate within the lexicon, even when much more complex structures are attested in a language (Rousset, 2004). On the other hand, typological measures capture the maximal syllable structure patterns in a language, which can be substantially and categorically complexified by processes of vowel reduction, including those associated with the phonetic erosion of grammatical morphemes. In that sense, the corpus-based syllable complexity measures are less appropriate than the typological measures for testing our specific hypotheses. However, they still provide a valuable point of comparison, given that other studies in this vein use Phonemes Per Syllable and similar metrics to measure syllable complexity (cf. Fenk-Oczlon, this issue).

It is important to note that while the global positive relationship between syllable complexity and morphological synthesis is statistically robust, the findings from the corpus study are not. This is a function of both the corpus study sample size and the relatively smaller diversity in syllable structures represented there. However, we note that given the size of the global correlation (r = 0.26–0.33), for a study of the design used here to have statistical power above the threshold of 0.8, it would require a sample of 84 corpora which are annotated and processed in roughly identical ways. This is an unrealistic possibility at present, especially with added considerations regarding genealogical and geographic diversity. Therefore, the findings presented here provide a good reference point for further investigations into this topic. Further, despite the statistical limitations of the data presented here, we consider it to be highly informative in that we know of no similarly deep study of local and part-of-speech synthesis patterns in such a diverse set of languages.

Although our findings are consistent with the predictions of a grammaticalization account, we find no direct evidence for grammaticalization being the driver of the positive correlation between syllable complexity and morphological synthesis. Indeed, investigating this in the language-specific detail required is far beyond the scope of the present work. We note that two of the languages in the sample, Beja and Movima, have complex codas only in the context of suffixation, a pattern which would be consistent with grammaticalization-related phonological reduction driving the direct overlapping of syllable complexity and synthesis. It has been found that as maximal syllable margin size increases, languages are more likely to have those maximal patterns only in morphologically complex contexts (Easterday, 2019). Perhaps in a sample with more diverse syllable complexity, such an analysis could be done alongside a deeper look at the diachronic development of consonantal grammatical morphemes and syllable structure complexity more generally within individual languages.

It is important to acknowledge, as discussed in section Grammaticalization: A Diachronic Source for a Positive Complexity Correlation? that the process of grammaticalization does not entail phonological reduction resulting in consonantal affixes, specifically, and may not entail much phonological reduction or morphological fusion at all (Schiering, 2010; Zingler, 2018). However, we would not necessarily expect a positive relationship between syllable complexity and morphological synthesis, especially at the local level, to emerge from phonologically or phonetically conditioned vowel reduction trajectories operating entirely independently of morphological considerations. As we have seen with the Lezgian and Mojeño Trinitario examples in section A Well-Motivated Positive Complexity Correlation, the outcomes of such patterns are quite variable, sometimes producing clusters across morpheme boundaries and sometimes not. While we are open to other explanations, we take the crosslinguistically common process of grammaticalization, and specifically its trajectories which produce consonantal morphemes, to be the most plausible candidate for a diachronic process targeting morphemes with direct effects on canonical syllable complexity. However, this remains a hypothesis.

Of course, there are many other important factors that we have not mentioned which may complicate our interpretations of the patterns observed. For example, synchronic patterns may obscure previously productive morphology such that phonological remnants are retained in the syllable patterns but the fossilized morphology is no longer analyzable. If such cases are frequent in any given sample, they may dampen the observed correlation between syllable complexity and morphological synthesis. On the other hand, languages with high syllable complexity metrics vary enormously in how prevalent those maximal structures are in the language. For those whose maximal syllable patterns are extremely marginal in both shape and distribution in the language (e.g., Katla), any correspondingly high index of synthesis cannot be regarded as theoretically well-motivated. In cases such as these, the complementary broad and deep approach taken here is especially valuable in that it allows for such confounds to be sorted out. Finally, it is important to recognize that the positive correlation established here is a tendency and has many exceptions. Notably, in both the typological survey and the corpus study, there are languages which have relatively low syllable complexity and relatively high morphological synthesis (e.g., Kalaallisut, Bora). Many languages do not have prosodic properties which favor vowel reduction (cf. Schiering, 2010), and in such cases there is little potential for a relationship between syllable complexity and morphological synthesis to develop.

Complexity correlations between the phonological and morphological subsystems of languages have been proposed for years. Although the usual approach is to seek out trade-offs, here we have offered empirical support for a positive relationship which crosses domain boundaries: syllable complexity and morphological synthesis. Further, we suggest that this correlation is not random, but the product of diachronic processes which have effects on both systems. Our study contributes a novel approach to investigations in this area, incorporating both broad typological sampling and deep corpus analysis. We hope that these findings and methodological contributions stimulate much further research into the intriguing area of positive complexity correlations.

## Data Availability Statement

The original contributions presented in the study are included in the article/Supplementary Material, further inquiries can be directed to the corresponding author.

## Author Contributions

All authors listed have made a substantial, direct and intellectual contribution to the work, and approved it for publication.

## Conflict of Interest

The authors declare that the research was conducted in the absence of any commercial or financial relationships that could be construed as a potential conflict of interest.

## Footnotes

^1^There is a process of rhythmic syncope, but this is limited to certain word structures, and in any case, does not affect the initial syllable of a word (Polian, 2013: 113–116).

^2^This resulted in very few removals, except for Movima (16% of word types) and Hoocak (10% of word types).

^3^While Southern Aymara has Highly Complex syllable structure, the precise number of consonants occurring in the maximal onset and coda could not be determined from the references consulted, so this language has been excluded from correlations using those values.

^4^Correlation coefficients are generally interpreted as follows: 0–0.1 = negligible, 0.1–0.39 = weak correlation, 0.4–0.6 = moderate correlation, 0.7–0.89 = strong correlation, 0.9–1 = strong correlation.

^5^Using robust regression also resulted in positive estimates. Further details about the output of robust regression are available in the Supplementary Materials.

## References

[B1] Auer P. (1993). Is a rhythm-based typology possible? A study of the role of prosody in phonological typology. KontRI Working Paper. Universität Konstanz, 21.

[B2] Bates D. Mächler M. Bolker B. Walker S. (2015). Fitting linear mixed-effects models using lme4. J. Stat. Softw. 67, 1–48. 10.18637/jss.v067.i01

[B3] Bentz C. Winter B. (2013). Languages with more second language learners tend to lose nominal case. Lang. Dyn. Change 3, 1–27. 10.1163/22105832-13030105

[B4] Bickel B. Nichols J. (2005). Inflectional synthesis of the verb, in The World Atlas of Language Structures, eds Haspelmath M. Dryer M. S. Gil D. Comrie B. (Oxford: Oxford University Press), 94–97.

[B5] Bisang W. (2004). Grammaticalization without coevolution of form and meaning: the case of tense-aspect-modality in East and Mainland Southeast Asia, in What Makes Grammaticalization? A Look from its Fringes and its Components [Trends in Linguistics, Studies and Monographs 158], eds Bisang W. Himmelmann N. P. Wiemer B. (Berlin: Mouton de Gruyter), 109–138.

[B6] Bürkner P.-C. (2017). brms: sn R package for Bayesian multilevel models using Stan. J. Stat. Softw. 80:1. 10.18637/jss.v080.i01

[B7] Bybee J. Perkins R. Pagliuca W. (1994). The Evolution of Grammar: Tense, Aspect, and Modality in the Languages of the World. Chicago: University of Chicago Press.

[B8] Chitoran I. Babaliyeva A. (2007). An acoustic description of high vowel syncope in Lezgian, in International Congress of Phonetic Sciences XVI (Melbourne), 2153–2156.

[B9] Clements G. N. (2003). Feature economy in sound systems. Phonology 2, 287–333. 10.1017/S095267570400003X

[B10] Coloma G. (2016). The existence of negative correlation between linguistic measures across languages. Corpus Linguist. Linguist. Theory 13, 1–26. 10.1515/cllt-2015-0020

[B11] Comrie B. (1989). Language Universals and Linguistic Typology. Chicago: University of Chicago Press.

[B11a] Cowell A. (2020). Arapaho DoReCo data set, in Language Documentation Reference Corpus (DoReCo) 1.0, eds Seifart F. Paschen L. Stave M. (Berlin; Lyon: Leibniz-Zentrum Allgemeine Sprachwissenschaft; Laboratoire Dynamique Du Langage UMR5596, CNRS; Université Lyon 2).

[B12] Delafontaine F. (2020). Multitool. San Francisco, CA: GitHub. Available online at: https://github.com/DoReCo/multitool (accessed 2020 September 3).

[B13] Díaz Montenegro E. (2019). El habla nasa (páez) de Munchique: nuevos acercamientos a su sociolingüística, fonología y sintaxis. dissertation. Lyon: Université Lumière Lyon 2.

[B14] Donegan P. J. Stampe D. (1983). Rhythm and the holistic organization of language structure, in Papers from the Parasession on the Interplay of Phonology, Morphology, and Syntax, eds Richardson J. F. Marks M. Chukerman A. (Chicago: CLS 1993), 337–353.

[B15] Dryer M. S. (1989). Large linguistic areas and language sampling. Stud. Lang. 13, 257–292. 10.1075/sl.13.2.03dry

[B16] Dryer M. S. (1992). The Greenbergian word order correlations. Language 68, 81–138. 10.1353/lan.1992.0028

[B17] Dryer M. S. Haspelmath M. (2013). The World Atlas of Language Structures Online. Leipzig: Max Planck Institute for Evolutionary Anthropology. Available online at http://wals.info (accessed December 6, 2020).

[B18] Easterday S. (2019). Highly Complex Syllable Structure: A Typological and Diachronic Study. (Studies in Laboratory Phonology 9). Berlin: Language Science Press.

[B19] Easterday S. Timm J. Maddieson I. (2011). The effects of phonological structure on the acoustic correlates of rhythm, in International Congress of Phonetic Sciences XVII (Hong Kong), 623–626.

[B20] Fenk A. Fenk-Oczlon G. (1993). Menzerath's law and the constant flow of linguistic information, in Contributions to Quantitative Linguistics: Proceedings of the First International Conference on Quantitative Linguistics, QUALICO, Trier, 1991, eds Köhler R. Rieger B. B. (Dordrecht: Kluwer), 11–31. 10.1007/978-94-011-1769-2_2

[B21] Fenk-Oczlon G. Fenk A. (2005). Crosslinguistic correlations between size of syllables, number of cases, and adposition order, in Sprache und Natürlichkeit. Gedenkband für Willi Mayerthaler, eds Fenk-Oczlon G. Winkler C. (Tübingen: Gunter Narr Verlag), 75–86.

[B22] Fenk-Oczlon G. Fenk A. (2008). Complexity trade-offs between the subsystems of language, in Language Complexity: Typology, Contact, Change, eds Miestamo M. Sinnemäki K. Karlsson F. (Amsterdam/Philadelphia: John Benjamins Publishing Company), 43–65. 10.1075/slcs.94.05fen

[B22a] Forker D. (2020). Sanzhi Dargwa DoReCo data set, in Language Documentation Reference Corpus (DoReCo) 1.0, eds Seifart F. Paschen L. Stave M. (Berlin; Lyon: Leibniz-Zentrum Allgemeine Sprachwissenschaft; Laboratoire Dynamique Du Langage UMR5596, CNRS; Université Lyon 2).

[B22b] Garcia-Laguia A. (2020). Northern Alta DoReCo data set, in Language Documentation Reference Corpus (DoReCo) 1.0, eds Seifart F. Paschen L. Stave M. (Berlin; Lyon: Leibniz-Zentrum Allgemeine Sprachwissenschaft; Laboratoire Dynamique Du Langage UMR5596, CNRS; Université Lyon 2).

[B23] Gil D. (1986). A prosodic typology of language. Folia Linguist 20, 165–231. 10.1515/flin.1986.20.1-2.165

[B24] Gordon M. K. (2016). Phonological Typology. Oxford: Oxford University Press. 10.1093/acprof:oso/9780199669004.001.0001

[B25] Greenberg J. (1954). A quantitative approach to the morphological typology of language, in Method and Perspective in Anthropology, ed Spencer R. F. (Minneapolis, MN: Univ of Minnesota Press), 192–220 (Reprinted in 1960 in International Journal of American Linguistics 26: 178–94). 10.1086/464575

[B25a] Gusev V. Klooster T. Wagner-Nagy B. Arkhipov A. (2020). Kamas DoReCo data set, in Language Documentation Reference Corpus (DoReCo) 1.0, eds Seifart F. Paschen L. Stave M. (Berlin; Lyon: Leibniz-Zentrum Allgemeine Sprachwissenschaft; Laboratoire Dynamique Du Langage UMR5596, CNRS; Université Lyon 2).

[B25b] Haig G. Vollmer M. Thiele H. (2020). Northern Kurdish DoReCo data set, in Language Documentation Reference Corpus (DoReCo) 1.0, eds Seifart F. Paschen L. Stave M. (Berlin; Lyon: Leibniz-Zentrum Allgemeine Sprachwissenschaft; Laboratoire Dynamique Du Langage UMR5596, CNRS; Université Lyon 2).

[B26] Hammarström H. Forkel R. Haspelmath M. Bank S. (2020). Glottolog 4.3. Jena: Max Planck Institute for the Science of Human History. Available online at: http://glottolog.org (accessed December 6, 2020).

[B26a] Hartmann I. (2013). Hoocak Corpus. Leipzig: MPI-EVA.

[B27] Haspelmath M. (1993). A grammar of Lezgian. (Mouton Grammar Library, 9). Berlin: Mouton de Gruyter. 10.1515/9783110884210

[B27a] Haude K. (2020). Movima DoReCo data set, in Language Documentation Reference Corpus (DoReCo) 1.0, eds Seifart F. Paschen L. Stave M. (Berlin; Lyon: Leibniz-Zentrum Allgemeine Sprachwissenschaft; Laboratoire Dynamique Du Langage UMR5596, CNRS; Université Lyon 2).

[B27b] Hellwig B. (2020a). Goemai DoReCo data set, in Language Documentation Reference Corpus (DoReCo) 1.0, eds Seifart F. Paschen L. Stave M. (Berlin; Lyon: Leibniz-Zentrum Allgemeine Sprachwissenschaft; Laboratoire Dynamique Du Langage UMR5596, CNRS; Université Lyon 2).

[B27c] Hellwig B. (2020b). Katla DoReCo data set, in Language Documentation Reference Corpus (DoReCo) 1.0, eds Seifart F. Paschen L. Stave M. (Berlin; Lyon: Leibniz-Zentrum Allgemeine Sprachwissenschaft; Laboratoire Dynamique Du Langage UMR5596, CNRS; Université Lyon 2).

[B28] Jolliffe I. (2002). Principal Component Analysis. New York, NY: Springer.

[B28a] Kazakevich O. Klyachko E. (2020). Evenki DoReCo data set, in Language Documentation Reference Corpus (DoReCo) 1.0, eds Seifart F. Paschen L. Stave M. (Berlin; Lyon: Leibniz-Zentrum Allgemeine Sprachwissenschaft; Laboratoire Dynamique Du Langage UMR5596, CNRS; Université Lyon 2).

[B29] Kuznetsova A. Brockhoff P. B. Christensen R. H. B. (2017). lmerTest package: tests in linear mixed effects models. J. Stat. Softw. 82:13. 10.18637/jss.v082.i13

[B30] Ladd D. R. Roberts S. G. Dediu D. (2015). Correlational studies in typological and historical linguistics. Ann. Rev. Linguist. 1, 221–241. 10.1146/annurev-linguist-030514-124819

[B31] Lindblom B. Maddieson I. (1988). Phonetic universals in consonant systems, in Language, Speech, and Mind: Studies in Honor of Victoria A. Fromkin, eds Hyman L. M. Fromkin V. Li C. N. (London: Taylor and Francis), 62–78.

[B32] Lüdecke D. (2020). sjPlot: Data Visualization for Statistics in Social Science. R package version 2.8.4. Available online at: https://CRAN.R-project.org/package=sjPlot (accessed December 6, 2020).

[B33] Maddieson I. (2006). Correlating phonological complexity: data and validation. Linguist. Typol. 10, 106–123. 10.1515/LINGTY.2006.017

[B34] Maddieson I. (2011). Phonological complexity in linguistic patterning, in Proceedings of the 17th International Congress of Phonetic Sciences (Hong Kong), 28–34.

[B34a] Meakins F. (2020). Gurindji Kriol DoReCo data set, in Language Documentation Reference Corpus (DoReCo) 1.0, eds Seifart F. Paschen L. Stave M. (Berlin; Lyon: Leibniz-Zentrum Allgemeine Sprachwissenschaft; Laboratoire Dynamique Du Langage UMR5596, CNRS; Université Lyon 2).

[B35] Nichols J. (2009). Linguistic complexity: a comprehensive definition and survey, in Linguistic Complexity as an Evolving Variable, eds Sampson G. Gil D. Trudgill P. (Oxford: Oxford University Press), 110–125.

[B36] Oh Y. M. Pellegrino F. Marsico E. Coupé C. (2013). A quantitative and typological approach to correlating linguistic complexity, in Proceedings of the 5th Conference on Quantitative Investigations in Theoretical Linguistics, 71.

[B37] Ohala J. J. (1979). Phonetic universals in phonological systems and their explanation. [Summary of symposium moderator's introduction.], in Proceedings of the 9th International Congress of Phonetic Sciences. Vol. 2. Copenhagen: Institute of Phonetics, 5–8.

[B38] Paschen L. Delafontaine F. Draxler C. Fuchs S. Stave M. Seifart F. (2020). Building a time-aligned cross-linguistic reference corpus from language documentation data (DoReCo), in Proceedings of The 12th Language Resources and Evaluation Conference, eds Calzolari N. Béchet F. Blache P. Choukri K. Cieri C. Declerck T.. (Marseille: European Language Resources Association), 2657–2666. Available online at: https://www.aclweb.org/anthology/2020.lrec-1.324.pdf (accessed December 6, 2020).

[B39] Plank F. (1998). The co-variation of phonology with morphology and syntax: a hopeful history. Linguist. Typol. 2, 195–230. 10.1515/lity.1998.2.2.195

[B40] Polian G. (2013). Gramática del tseltal de Oxchuc. Ciudad de México: Centro de Investigaciones y Estudios Superiores en Antropología Social.

[B41] R Core-Team (2020). R: A Language and Environment for Statistical Computing. Vienna: R Foundation for Statistical Computing Available online at: https://www.R-project.org/ (accessed December 6, 2020).

[B42] Ramus F. Nespor M. Mehler J. (1999). Correlates of linguistic rhythm in the speech signal. Cognition 73, 265–292. 10.1016/S0010-0277(99)00058-X 10585517

[B42a] Ring H. (2020). Pnar DoReCo data set, in Language Documentation Reference Corpus (DoReCo) 1.0, eds Seifart F. Paschen L. Stave M. (Berlin; Lyon: Leibniz-Zentrum Allgemeine Sprachwissenschaft; Laboratoire Dynamique Du Langage UMR5596, CNRS; Université Lyon 2).

[B43] Rose F. (2019). Rhythmic syncope and opacity in Mojeño Trinitario. Phonol. Data Anal. 1, 1–25. 10.3765/pda.v1art2.2

[B44] Rose F. (2020). Mojeño Trinitario DoReCo data set, in Language Documentation Reference Corpus (DoReCo) 1.0, eds Seifart F. Paschen L. Stave M. (Berlin; Lyon: Leibniz-Zentrum Allgemeine Sprachwissenschaft; Laboratoire Dynamique Du Langage UMR5596, CNRS; Université Lyon 2).

[B45] Rousset I. (2004). Structures syllabiques et lexicales des langues du monde. dissertation. Grenoble: Université Grenoble.

[B46] Saporta S. (1963). Phoneme distribution and language universals, in Universals of Language, ed Greenberg J. H. (Cambridge: MIT Press), 61–67.

[B47] Schiering R. (2007). The phonological basis of linguistic rhythm: cross-linguistic data and diachronic interpretation. Sprachtypologie Universalienforschung 60, 337–359. 10.1524/stuf.2007.60.4.337

[B48] Schiering R. (2010). Reconsidering erosion in grammaticalization, in Grammaticalization: Current Views and Issues. Studies in Language Companion Series 119, ed Stathi K. Gehweiler E. König E. (Amsterdam/Philadelphia: John Benjamins), 73–100. 10.1075/slcs.119.06sch

[B49] Schloerke B. Cook D. Larmarange J. Briatte F. Marbach M. Thoen E. (2020). GGally: Extension to “ggplot2”. R package version 2.0.0. Available online at: https://CRAN.R-project.org/package=GGally (accessed December 6, 2020).

[B49a] Seifart F. (2020). Bora DoReCo data set, in Language Documentation Reference Corpus (DoReCo) 1.0, eds Seifart F. Paschen L. Stave M. (Berlin; Lyon: Leibniz-Zentrum Allgemeine Sprachwissenschaft; Laboratoire Dynamique Du Langage UMR5596, CNRS; Université Lyon 2).

[B50] Shosted R. K. (2006). Correlating complexity: a typological approach. Linguist. Typol. 10, 1–40. 10.1515/LINGTY.2006.001

[B51] Sinnemäki K. (2008). Complexity trade-offs in core argument marking, in Language Complexity: Typology, Contact, Change, eds Miestamo M. Sinnemäki K. Karlsson F. (Amsterdam/Philadelphia: John Benjamins), 67–88. 10.1075/slcs.94.06sin

[B52] Sinnemäki K. (2019). On the distribution and complexity of gender and numeral classifiers, in Grammatical Gender and Linguistic Complexity, eds Di Garbo F. Olsson B. Walchli B. (Berlin: Language Science Press), 133–200.

[B53] Sinnemäki K. Di Garbo F. (2018). Language structures may adapt to the sociolinguistic environment, but it matters what and how you count: a typological study of verbal and nominal complexity. Front. Psychol. 9:1141. 10.3389/fpsyg.2018.01141 30154738 PMC6102949

[B53a] Skopeteas S. Moisidi V. Tsetereli N. Lorenz J. Schröter S. (2020). Urum DoReCo data set, in Language Documentation Reference Corpus (DoReCo) 1.0, eds Seifart F. Paschen L. Stave M. (Berlin; Lyon: Leibniz-Zentrum Allgemeine Sprachwissenschaft; Laboratoire Dynamique Du Langage UMR5596, CNRS; Université Lyon 2).

[B54] Slowikowski K. (2019). ggrepel: Automatically position non-overlapping text labels with ggplot2. R package version 0.8.1. Available online at: https://CRAN.R-project.org/package$=$ggrepel (accessed December 6, 2020).

[B55] Stevens K. N. (1989). On the quantal nature of speech. J. Phon. 17, 3–45. 10.1016/S0095-4470(19)31520-7

[B56] Strunk J. Schiel F. Seifart F. (2014). Untrained forced alignment of transcriptions and audio for language documentation corpora using WebMAUS, in LREC (Reykjavik), 3940–3947. Available online at: http://www.lrec-conf.org/proceedings/lrec2014/pdf/1176_Paper.pdf (accessed December 6, 2020).

[B57] Tang Y. Horikoshi M. Li W. (2016). ggfortify: unified interface to visualize statistical result of popular R packages. R. J. 8, 474–485. 10.32614/RJ-2016-060

[B57a] Teo A. Kinny H. S. (2020). Sümi DoReCo data set, in Language Documentation Reference Corpus (DoReCo) 1.0, eds Seifart F. Paschen L. Stave M. (Berlin; Lyon: Leibniz-Zentrum Allgemeine Sprachwissenschaft; Laboratoire Dynamique Du Langage UMR5596, CNRS; Université Lyon 2). Available online at: https://hdl.handle.net/11280/545e9666.

[B57b] Thieberger N. (2020). Nafsan DoReCo data set, in Language Documentation Reference Corpus (DoReCo) 1.0, eds Seifart F. Paschen L. Stave M. (Berlin; Lyon: Leibniz-Zentrum Allgemeine Sprachwissenschaft; Laboratoire Dynamique Du Langage UMR5596, CNRS; Université Lyon 2).

[B58] Traugott E. C. (2002). From etymology to historical pragmatics, in Studies in the History of the English Language, eds Minkova D. Stockwell R. (Berlin/New York: Mouton de Gruyter), 19–49. 10.1515/9783110197143.1.19

[B58a] Vanhove M. (2020). Beja DoReCo data set, annotated within CorpAfroAs and CORPORAN, reannotated within DORECO, in Language Documentation Reference Corpus (DoReCo) 1.0, eds Seifart F. Paschen L. Stave M. (Berlin; Lyon: Leibniz-Zentrum Allgemeine Sprachwissenschaft; Laboratoire Dynamique Du Langage UMR5596, CNRS; Université Lyon 2).

[B58b] Vydrina A. (2020). Kakabe DoReCo data set, in Language Documentation Reference Corpus (DoReCo) 1.0, eds Seifart F. Paschen L. Stave M. (Berlin; Lyon: Leibniz-Zentrum Allgemeine Sprachwissenschaft; Laboratoire Dynamique Du Langage UMR5596, CNRS; Université Lyon 2).

[B58c] Wegener C. (2020). Savosavo DoReCo data set, in Language Documentation Reference Corpus (DoReCo) 1.0, eds Seifart F. Paschen L. Stave M. (Berlin; Lyon: Leibniz-Zentrum Allgemeine Sprachwissenschaft; Laboratoire Dynamique Du Langage UMR5596, CNRS; Université Lyon 2).

[B59] Wickham H. (2017). tidyverse: easily install and load the Tidyverse. R package version 1.2.1. Available online at: https://CRAN.R-project.org/package=tidyverse (accessed December 6, 2020).

[B60] Wickham H. Bryan J. (2019). readxl: Read Excel files. R package version 1.3.1. Available online at: https://CRAN.R-project.org/package=readxl (accessed December 6, 2020).

[B61] Wickham H. Seidel D. (2020). scales: Scale Functions for Visualization. R package version 1.1.1. Available online at: https://CRAN.R-project.org/package=scales (accessed December 6, 2020).

[B61a] Witzlack-Makarevich A. Namyalo S. Kiriggwajjo A. Molochieva Z. Atuhairwe A. (2020). Ruuli DoReCo data set, in Language Documentation Reference Corpus (DoReCo) 1.0, eds Seifart F. Paschen L. Stave M. (Berlin; Lyon: Leibniz-Zentrum Allgemeine Sprachwissenschaft; Laboratoire Dynamique Du Langage UMR5596, CNRS; Université Lyon 2).

[B62] Zingler T. (2018). Reduction without fusion: grammaticalization and wordhood in Turkish. Folia Linguist. 52, 415–447. 10.1515/flin-2018-0011

