# Corrigendum: Syllable Complexity and Morphological Synthesis: A Well-Motivated Positive Complexity Correlation Across Subdomains

**DOI:** 10.3389/fpsyg.2021.689645

**Published:** 2021-05-26

**Authors:** Shelece Easterday, Matthew Stave, Marc Allassonnière-Tang, Frank Seifart

**Affiliations:** ^1^Department of Linguistics, University of Hawai'i Mãnoa, Honolulu, HI, United States; ^2^UMR 5596 Dynamique du Langage, Université Lumière Lyon 2 and Centre National de la Recherche Scientifique, Lyon, France; ^3^Leibniz-Centre General Linguistics (ZAS), Berlin, Germany

**Keywords:** complexity correlations, syllable structure, morphological synthesis, grammaticalization, language change, linguistic typology, corpus study

In the original article, there was an error. The acknowledgments were not included. The following Acknowledgments section has been added and the additional references have been added to the reference list.

**Acknowledgments**

The authors are thankful to the following experts for sharing their data with the DoReCo project, which was used in the corpus analyses presented in the current paper (see Table 2 of the supplementary materials for details about their data contributions and full bibliographical references): Andrew Cowell (Arapaho, Cowell, 2020); Diana Forker (Sanzhi Dargwa, Forker, 2020); Alexandro Garcia-Laguia (Northern Alta, Garcia-Laguia, 2020); Valentin Gusev, Tiina Klooster, Beáta Wagner-Nagy, and Alexandre Arkhipov (Kamas, Gusev et al., 2020); Geoffrey Haig, Maria Vollmer, and Hanna Thiele (Northern Kurdish, Haig et al., 2020); Iren Hartmann (Hoocak, Hartmann, 2013); Katharina Haude (Movima, Haude, 2020); Birgit Hellwig (Goemai and Katla, Hellwig, 2020a,b); Olga Kazakevich and Elena Klyachko (Evenki, Kazakevich and Klyachko, 2020); Felicity Meakins (Gurindji Kriol, Meakins, 2020); Hiram Ring (Pnar, Ring, 2020); Françoise Rose (Mojeño Trinitario, Rose, 2020); Frank Seifart (Bora, Seifart, 2020); Stavros Skopeteas, Violeta Moisidi, Nutsa Tsetereli, Johanna Lorenz, and Stefanie Schröter (Urum, Skopeteas et al., 2020); Amos Teo and H. Salome Kinny (Sümi, Teo and Kinny, 2020); Nick Thieberger (Nafsan, Thieberger, 2020); Martine Vanhove (Beja, Vanhove, 2020); Alexandra Vydrina (Kakabe, Vydrina, 2020); Claudia Wegener (Savosavo, Wegener, 2020); Alena Witzlack-Makarevich, Saudah Namyalo, Anatol Kiriggwajjo, Zarina Molochieva, and Amos Atuhairwe (Ruuli, Witzlack-Makarevich et al., 2020).

The authors apologize for this error and state that this does not change the scientific conclusions of the article in any way. The original article has been updated.
