# Supplementary material for: Syllable Complexity and Morphological Synthesis: A Well-Motivated Positive Complexity Correlation Across Subdomains
Source: Front Psychol. 2021 Mar 17;12:638659. doi: 10.3389/fpsyg.2021.638659 (PMC8010299; doi:10.3389/fpsyg.2021.638659)
Supplement: Supplementary file 1 [file Table_1.DOCX]

**6.1. Appendix A: Language sample, typological survey**

|  |  |  | **Syllable structure complexity measures** | | | **Index of synthesis measures** | |  |
| --- | --- | --- | --- | --- | --- | --- | --- | --- |
| **Language** | **WALS genus** | **Glottocode** | **(1)** | **(2)** | **(3)** | **N morphemes** | **N words** | **Reference** |
| Passamaquoddy- Maliseet | Algonquian | **male1292** | HC | 6 | 6 | 262 | 125 | Leavitt (1996) |
| Menya | Angan | **meny1245** | HC | 4 | 4 | 745 | 301 | Whitehead (2004) |
| Madi | Arauan | **jama1261** | S | 1 | 1 | 682 | 483 | Dixon (2004) |
| Witsuwit’en | Athapaskan | **wits1234** | C | 4 | 4 | 316 | 132 | Hargus (2007) |
| Southern Aymara | Aymaran | **sout2996** | HC | N/A | N/A | 606 | 227 | Coler (2014) |
| Sre (Kơho) | Bahnaric | **koho1244** | C | 5 | 4 | 90 | 89 | Olsen (2014) |
| Basque | Basque | **basq1248** | C | 4 | 3 | 462 | 284 | Hualde & Urbina (2003) |
| Tashlhiyt | Berber | **tach1250** | HC | 2 | 2 | 144 | 76 | Ridouane (2014) |
| Bilua | Bilua | **bilu1245** | S | 1 | 1 | 505 | 314 | Obata (2003) |
| Bororo | Bororoan | **boro1282** | S | 1 | 1 | 531 | 332 | Nonato (2008) |
| Yi (Nuosu) | Burmese-Lolo | **sich1238** | S | 1 | 1 | 465 | 455 | Gerner (2013) |
| Wichita | Caddoan | **wich1260** | HC | 8 | 8 | 415 | 152 | Rood (1976) |
| Carib | Cariban | **gali1262** | MC | 2 | 2 | 619 | 353 | Courtz (2008) |
| Tukang Besi North | Celebic | **tuka1248** | S | 1 | 1 | 605 | 398 | Donohue (1999) |
| Tehuelche | Chon Proper | **tehu1242** | HC | 5 | 4.5 | 412 | 249 | Fernandez Garay & Hernandez (2006) |
| Lakota | Core Siouan | **lako1247** | C | 3 | 3 | 282 | 215 | Ingham (2003) |
| Igala | Defoid | **igal1242** | S | 1 | 1 | 317 | 215 | Ejeba (2017) |
| Dizin (Central) | Dizoid | **dizi1235** | C | 4 | 3.5 | 485 | 251 | Beachy (2005) |
| Toro So | Dogon | **toro1252** | S | 2 | 1.5 | 339 | 276 | Plungian (1995) |
| Duna | Duna | **duna1248** | MC | 2 | 1.5 | 577 | 344 | San Roque (2008) |
| Gaam | Eastern Jebel | **gaam1241** | C | 3 | 2.5 | 503 | 339 | Stirtz (2011) |
| Mann | Eastern Mande | **mann1248** | S | 1 | 1 | 511 | 499 | Khachaturyan (2014) |
| East Kewa | Engan | **east2516** | S | 1 | 1 | 399 | 278 | Franklin & Franklin (1978) |
| Kalaallisut | Eskimo | **kala1399** | MC | 2 | 2 | 471 | 156 | Sadock (2003) |
| Yau | Finisterre-Huon | **yaum1237** | MC | 3 | 2.5 | 492 | 198 | Sarvasy (2014) |
| Fur | Fur | **furr1244** | MC | 2 | 2 | 234 | 202 | Jakobi (1990) |
| Apinayé | Ge-Kaingang | **apin1244** | C | 4 | 3.5 | 445 | 409 | Oliveira (2005) |
| Western Xiangxi Miao | Hmong-Mien | **west2430** | S | 1 | 1 | 202 | 168 | Sposato (2015) |
| San Dionisio del Mar Huave | Huavean | **sand1278** | MC | 3 | 2.5 | 406 | 260 | Salminen (2016) |
| Murui Huitoto | Huitoto | **muru1274** | S | 1 | 1.5 | 729 | 377 | Wojtylak (2017) |
| Darai | Indic | **dara1250** | MC | 3 | 2.5 | 734 | 472 | Dhakal (2012) |
| Thompson | Interior Salish | **thom1243** | HC | 9 | 9 | 506 | 297 | Thompson & Thompson (1992) |
| Itonama | Itonama | **iton1250** | C | 3 | 3 | 805 | 324 | Crevels (2012) |
| Aguaruna | Jivaroan | **agua1253** | C | 3 | 2.5 | 586 | 268 | Overall (2007) |
| Lao | Kam-Tai | **laoo1244** | MC | 2 | 2 | 381 | 362 | Enfield (2007) |
| Karok | Karok | **karo1304** | MC | 2 | 2 | 614 | 252 | Angulo & Freeland (1931) |
| Georgian | Kartvelian | **nucl1302** | HC | 13 | 12 | 594 | 246 | Hewitt (1995) |
| Pacoh | Katuic | **paco1243** | MC | 3 | 2.5 | 557 | 520 | Watson (1980) |
| Katukína-Kanamarí | Katukinan | **kana1291** | MC | 2 | 2 | 426 | 294 | Ishy de Magalhaes (2018) |
| Jemez | Kiowa-Tanoan | **jeme1245** | S | 2 | 2 | 408 | 244 | Yumitani (1998) |
| Grass Koiari | Koiarian | **gras1249** | S | 1 | 1 | 488 | 318 | Dutton (1996) |
| Kuot | Kuot | **kuot1243** | MC | 2 | 2 | 466 | 280 | Lindström (2002) |
| Ngiti | Lendu | **ngit1239** | S | 1 | 1 | 571 | 415 | Kutsch-Lojenga (1994) |
| Lepcha | Lepcha | **lepc1244** | C | 4 | 3.5 | 249 | 144 | Plaisier (2007) |
| Lezgian | Lezgic | **lezg1247** | HC | 5 | 5 | 676 | 425 | Haspelmath (1993) |
| Maba | Maban | **maba1277** | MC | 2 | 2 | 296 | 140 | Weiss (2009) |
| Chepang | Mahakiranti | **chep1245** | C | 5 | 4 | 678 | 321 | Caughley (1982) |
| Kamasau | Marienberg | **kama1367** | MC | 3 | 2.5 | 639 | 455 | Sanders & Sanders (1994) |
| Nivaclé | Matacoan | **niva1238** | C | 3 | 3 | 407 | 234 | Fabre (2016) |
| Aguacatenango Tzeltal | Mayan | **tzel1254** | C | 4 | 3.5 | 446 | 299 | Polian (2006) |
| Highland Popoluca | Mixe-Zoque | **high1276** | HC | 5 | 4.5 | 566 | 286 | De Jong Boudreault (2009) |
| Pinotepa Mixtec | Mixtecan | **pino1237** | S | 1 | 1 | 382 | 271 | Costello (2014) |
| Tu | Mongolic | **tuuu1240** | MC | 3 | 2 | 547 | 377 | Slater (2003) |
| Kharia | Munda | **khar1287** | MC | 2 | 2 | 604 | 399 | Peterson (2011) |
| Choctaw | Muskogean | **choc1276** | MC | 2 | 2 | 552 | 263 | Broadwell (2006) |
| Hup | Nadahup | **hupd1244** | MC | 2 | 2 | 600 | 273 | Epps (2008) |
| Nimboran | Nimboran | **nucl1633** | C | 4 | 3.5 | 334 | 198 | May (1997) |
| Nivkh (W. Sakhalin) | Nivkh | **gily1242** | C | 5 | 4.5 | 408 | 240 | Gruzdeva (1998) |
| Tobelo | North Halmaheran | **tobe1252** | S | 1 | 1 | 539 | 271 | Holton (2003) |
| Bench | North Omotic | **benc1235** | HC | 5 | 4.5 | 594 | 261 | Rapold (2006) |
| Maybrat | North-Central Bird’s Head | **maib1239** | MC | 2 | 2 | 689 | 453 | Dol (2007) |
| Kabardian | Northwest Caucasian | **kaba1278** | HC | 5 | 5 | 571 | 229 | Applebaum (2013) |
| Ute | Numic | **utes1238** | S | 2 | 2 | 593 | 255 | Givón (2013) |
| Bardi | Nyulnyulan | **bard1255** | C | 3 | 2.5 | 307 | 151 | Bowern (2012) |
| Lelepa | Oceanic | **lele1267** | C | 5 | 4 | 586 | 406 | Lacrampe (2014) |
| Nakanai | Oceanic | **naka1262** | S | 1 | 1 | 498 | 406 | Johnston (1980) |
| Oksapmin | Oksapmin | **oksa1245** | C | 3 | 3 | 843 | 482 | Loughnane (2009) |
| Paiwan | Paiwan | **paiw1248** | MC | 2 | 2 | 544 | 433 | Chang (2006) |
| Pech | Paya | **pech1241** | C | 4 | 3 | 196 | 69 | Holt (1999) |
| Apurinã | Purus | **apur1254** | S | 1 | 1 | 714 | 347 | Facundes (2000) |
| Yine | Purus | **yine1238** | HC | 3 | 3 | 539 | 257 | Hanson (2010) |
| Quechua (Imbabura) | Quechuan | **imba1240** | MC | 2 | 2 | 206 | 97 | Carpenter (1982) |
| Sahaptin (Yakama) | Sahaptian | **yaki1237** | HC | 8 | 7.5 | 575 | 324 | Jansen (2010) |
| Sandawe | Sandawe | **sand1273** | MC | 3 | 2 | 777 | 406 | Steeman (2011) |
| Savosavo | Savosavo | **savo1255** | S | 1 | 1 | 630 | 396 | Wegener (2008) |
| Alamblak | Sepik Hill | **alam1246** | HC | 6 | 5.5 | 1264 | 502 | Bruce (1984) |
| Seri | Seri | **seri1257** | HC | 8 | 8 | 643 | 360 | Marlett (1981) |
| Ma'ya | South Halmahera - West New Guinea | **maya1282** | C | 3 | 3 | 564 | 449 | Van der Leeden (1993) |
| Itelmen | Southern Chukotko-Kamchatkan | **itel1242** | HC | 12 | 11 | 876 | 438 | Georg & Volodin (1999) |
| Nuu-chah-nulth | Southern Wakashan | **nuuc1236** | HC | 5 | 5 | 545 | 212 | Stonham (1999) |
| Cavineña | Tacanan | **cavi1250** | S | 2 | 2 | 535 | 309 | Guillaume (2008) |
| Teribe | Talamanca | **teri1250** | HC | 5 | 4.5 | 595 | 522 | Quesada (2000) |
| Tohono O’odham | Tepiman | **toho1245** | HC | 8 | 8 | 353 | 250 | Saxton (1982) |
| Huehuetla Tepehua | Totonacan | **hueh1236** | HC | 5 | 5 | 623 | 387 | Kung (2007) |
| Saaroa | Tsou | **saar1237** | S | 2 | 1.5 | 493 | 288 | Pan (2012) |
| Cocama-Cocamilla | Tupi-Guaraní | **coca1259** | MC | 3 | 2 | 489 | 329 | Vallejos (2010) |
| Eastern Khanty | Ugric | **khan1273** | MC | 3 | 2.5 | 649 | 342 | Filchenko (2007) |
| Urarina | Urarina | **urar1246** | S | 1 | 1 | 270 | 169 | Olawsky (2006) |
| Chipaya | Uru-Chipaya | **chip1262** | C | 4 | 4 | 342 | 161 | Cerrón-Palomino (2006) |
| Warao | Warao | **wara1303** | S | 1 | 1 | 182 | 116 | Romero-Figeroa (1997) |
| Rotokas | West Bougainville | **roto1249** | S | 1 | 1 | 642 | 293 | Robinson (2011) |
| Wutung | Western Skou | **wutu1244** | HC | 5 | 4.5 | 340 | 313 | Marmion (2010) |
| Ngarinyin | Worrorran | **ngar1284** | C | 5 | 4 | 1142 | 573 | Coate & Oates (1970) |
| Ket | Yeniseian | **kett1243** | C | 5 | 5 | 602 | 267 | Vajda (2004) |
| Yuchi | Yuchi | **yuch1247** | C | 3 | 3 | 316 | 142 | Linn (2001) |

**References, Appendix A**

Angulo, J. de and Freeland, L.S. 1931. Karok texts. International Journal of American Linguistics, 6(3/4): 194-226.

Applebaum, A. B. 2013. Prosody and grammar in Kabardian. [dissertation]. [Santa Barbara]: University of California Santa Barbara.

Beachy, M. D. 2005. An overview of Central Dizin phonology and morphology. [M.A. thesis]. [Arlington]: University of Texas at Arlington.

Bowern, C. 2012. A grammar of Bardi. Berlin: Walter de Gruyter.

Broadwell, G. A. 2006. A Choctaw reference grammar. Lincoln: University of Nebraska Press.

Bruce, L. P. 1984. The Alamblak Language of Papua New Guinea (East Sepik). (Pacific Linguistics, Series C, No. 81). Canberra: Research School of Pacific Studies, Australian National University.

Carpenter, L. K. 1982. Equadorian Quichua: descriptive sketch and variation [dissertation]. [Gainesville]: University of Florida, Gainesville.

Caughley, R. C. 1982. Syntax and Morphology of the Verb in Chepang. (Pacific Linguistics: Series B, 84.) Canberra: Research School of Pacific and Asian Studies, Australian National University.

Cerrón-Palomino, R. 2006. El chipaya o la lengua de los hombres del agua. Lima: Pontificia Universidad Católica del Perú.

Chang, A. H. 2006. A reference grammar of Paiwan. [dissertation]. [Canberra]: The Australian National University.

Coate, H. H. J. and Oates, L. 1970. A grammar of Ngarinjin. Canberra: Australian Institute of Aboriginal Studies.

Coler, M. 2014. A Grammar of Muylaq' Aymara: Aymara as spoken in Southern Peru. Leiden: Brill.

Costello, R. A. 2014. Aspect and mood in Jicaltepec Mixtec. [M.A. thesis]. [Dallas]: The Graduate Institute of Applied Linguistics.

Courtz, H. 2008. A Carib grammar and dictionary. Toronto: Magoria Books.

Crevels, M. 2012. “Itonama,” in Ambito Andino, ed. Crevels, M. and Muysken, P. (La Paz: Plural Editores), 233-294.

De Jong Boudreault, L. J. 2009. A grammar of Sierra Popoluca (Soteapanec, a Mixe-Zoquean language). [dissertation]. [Austin]: University of Texas at Austin.

Dhakal, D. N. 2012. Darai grammar. München: Lincom Europa.

Dixon, R. M. W. 2004. The Jarawara Language of Southern Amazonia. Oxford: Oxford University Press.

Dol, P. 2007. A grammar of Maybrat: a language of the Bird's Head Pennisula, Papua Province, Indonesia. Canberra: Pacific Linguistics.

Donohue, M. 1999. A Grammar of Tukang Besi. Berlin/New York: Mouton de Gruyter.

Dutton, T. E. 1996. Koiari. München: Lincom Europa.

Ejeba, S. 2017. A grammar of Igala. Port Harcourt: M & J Grand Orbit Communications Ltd.

Enfield, N. J. 2007. A grammar of Lao. Berlin: Mouton de Gruyter.

Epps, P. 2008. A grammar of Hup. Berlin/New York: Mouton de Gruyter.

Fabre, A. 2016. Gramática de la lengua Nivacle (familia Mataguayo, Chaco Paraguayo). (LINCOM Studies in Native American Linguistics, 78.) München: LINCOM.

Facundes, S. da Silva. 2000. The language of the Apurinã people of Brazil (Maipure/Arawak). [dissertation]. [Buffalo]: State University of New York, Buffalo.

Fernández Garay, A. Hernández, G. 2006. Textos tehuelches (aonek’o ʔaʔjen). München: Lincom Europa.

Filchenko, A. Y. 2007. A grammar of Eastern Khanty. [dissertation]. [Houston]: Rice University.

Franklin, K. J. and Franklin, J. 1978. A Kewa dictionary, with supplementary grammatical and anthropological materials. (Pacific Linguistics, Series C, 53.) Canberra: Australian National University.

Georg, S. and Volodin, A. P. 1999. Die itelmenische Sprache: Grammatik und Texte. Weisbaden: Harrassowitz Verlag.

Gerner, M. 2013. A grammar of Nuosu. Berlin/Boston: De Gruyter Mouton.

Givón, T. 2013. Ute Texts. (Culture and Language Use, 7.) Amsterdam: John Benjamins.

Gruzdeva, E. 1998. Nivkh. München and Newcastle: Lincom Europa.

Guillaume, A. 2008. A grammar of Cavineña. (Mouton Grammar Library, 44.) Berlin: Mouton de Gruyter.

Hanson, R. 2010. A grammar of Yine (Piro). [dissertation]. [Melbourne]: La Trobe University.

Hargus, S. 2007. Witsuwit'en grammar: phonetics, phonology, morphology. (First Nations Languages.) Vancouver: UBC Press.

Haspelmath, M. 1993. A grammar of Lezgian. (Mouton Grammar Library, 9.) Berlin: Mouton de Gruyter.

Hewitt, B.G. 1995. Georgian: A structural reference grammar. Amsterdam: John Benjamins.

Holt, D. 1999. Pech (Paya). München: Lincom Europa.

Holton, G. 2003. Tobelo. (Languages of the World/Materials, 328.) Munich: München: Lincom.

Hualde, J. I. and Ortiz de Urbina, J. (eds.). 2003. *A grammar of Basque.* Berlin/New York: Mouton de Gruyter.

Ingham, B. 2003. Lakota. (Languages of the World: Materials, 426.) München: Lincom.

Ishy de Magalhães, P. H. 2018. Kanamari do Juruá (família Katukina): aspectos fonológicos e morfosintáticos. [dissertation]. [Campinas]: Universidade Estadual de Campinas.

Jakobi, A. 1990. A Fur Grammar: Phonology, Morphophonology, and Morphology. Hamburg: Helmut Buske Verlag.

Jansen, J. 2010. A grammar of Yakima Ichishkíin/Sahaptin. [dissertation]. Eugene: University of Oregon.

Johnston, R. L. 1980. Nakanai of New Britain: the Grammar of an Oceanic Language. (Pacific Linguistics: Series B, 70.) Canberra: Research School of Pacific and Asian Studies, Australian National University.

Khachaturyan, M. 2014. Grammaire de la langue Mano (Mandé-Sud) dans une perspective typologique. [dissertation]. [Paris]: Institut National des Langues et Civilisations Orientales (INALCO).

Kung, S. S. 2007. A descriptive grammar of Huehuetla Tepehua. Ann Arbor: Austin: University of Texas. [dissertation]. [Austin]: University of Texas at Austin.

Kutsch Lojenga, C., and Waag, C. 2004. “The sounds and tones of Fur,” in Occasional Papers in the Study of Sudanese Languages 9, ed. L. G. Gilley (Entebbe: SIL Sudan), 1–25.

Lacrampe, S. 2014. Lelepa: Topics in the grammar of a Vanuatu language. [dissertation]. [Canberra]: Australian National University.

Leavitt, R. M. 1996. Passamaquoddy-Maliseet. München: Lincom Europa.

Lindström, E. 2002. Topics in the Grammar of Kuot. [dissertation]. [Stockholm]: Stockholm University.

Linn, M. S. 2001. A grammar of Euchee (Yuchi). [dissertation]. [Lawrence]: University of Kansas.

Loughnane, R. 2009. A grammar of Oksapmin. [dissertation]. [Melbourne]: The University of Melbourne.

Marlett, S. A. 1981. The Structure of Seri. [dissertation]. [San Diego]: University of California, San Diego.

Marmion, D. 2010. Topics in the phonology and morphology of Wutung. [dissertation]. [Canberra]: The Australian National University.

May, K. 1997. A study of the Nimboran language: phonology, morphology, and phrase structure. [M.A. thesis]. [Melbourne]: La Trobe University.

Nonato, R. B. 2008. Ainore Boe egore: um estudo descritivo da língua bororo. Campinas: Univ. [M.A. thesis]. [Campinas]: Universidade Estadual de Campinas.

Obata, K. 2003. A Grammar of Bilua. Canberra: Pacific Linguistics.

Olawsky, K. J. 2006. A grammar of Urarina. New York: Mouton de Gruyter.

Oliveira, C. Cunha de. 2005. The language of the Apinajé people of central Brazil. [dissertation]. [Eugene]: University of Oregon.

Olsen, N. H. 2014. A descriptive grammar of Koho-Sre: A Mon-Khmer language. [dissertation]. [Salt Lake City]: University of Utah.

Overall, S. 2007. A grammar of Aguaruna. [dissertation]. [Bundoora]: LaTrobe University.

Pan, C. 2012. A Grammar of Lha'alua, an Austronesian Language of Taiwan. [dissertation]. [Townsville]: James Cook University.

Peterson, J. 2011. A grammar of Kharia: A South Munda language. Leiden/Boston: Brill.

Plaisier, H. 2007. A grammar of Lepcha. Leiden/Boston: Brill.

Plungian, V. 1995. Dogon. (Languages of the World/Materials, 64.) München: Lincom.

Polian, G. 2006. Eléments de grammaire du tseltal, une langue maya du Mexique. Paris: L’Harmattan.

Quesada, J. D. 2000. A Grammar of Teribe. (LINCOM Studies in Native American Linguistics, 36.) München: Lincom.

Rapold, C. 2006. Towards a grammar of Benchnon. [dissertation]. [Leiden]: Rijksuniversiteit te Leiden.

Ridouane, R. 2014. Tashlhiyt Berber. Journal of the International Phonetic Association, 44(2): 207-221.

Robinson, S. 2011. Split intransitivity in Rotokas, a Papuan language of Bougainville. [dissertation]. [Nijmegen]: Radboud University.

Romero-Figeroa, A. 1997. A reference grammar of Warao. München: Lincom Europa.

Rood, D. S. 1976. Wichita Grammar. (Garland studies in American Indian linguistics.) New York: Garland.

Sadock, J. M. 2003. A Grammar of Kalaallisut (West Greenlandic Inuttut). (Languages of the World/Materials, 162.) München: Lincom.

Salminen, M. B. 2016. A grammar of Umbeyajts as spoken by the Ikojts people of San Dionisio del Mar, Oaxaca, Mexico. [dissertation]. [Townsville]: James Cook University.

San Roque, L. 2008. An introduction to Duna grammar. [dissertation]. [Canberra]: Australian National University.

Sanders, A. G. and Sanders, J. 1994. Kamasau (Wand Tuan) Grammar: Morpheme to Discourse. [Unpublished ms.]

Sarvasy, H. S. 2014. A grammar of Nungon, a Papuan language of Morobe Province, Papua New Guinea. [dissertation]. [Townsville]: James Cook University.

Saxton, D. 1982. “Papago,” in Uto-Aztecan Grammatical Sketches, ed. R. Langacker (Dallas: Summer Institute of Linguistics), 93-266.

Slater, K. W. 2003. A grammar of Mangghuer: a Mongolic language of China's Qinghai-Gansu Sprachbund. London: Routledge Curzon.

Sposato, A. 2015. A grammar of Xong. [dissertation]. [Buffalo]: State University of New York at Buffalo.

Steeman, S. 2012. A grammar of Sandawe: A Khoisan language of Tanzania. 295. [dissertation]. [Leiden]: Rijksuniversiteit te Leiden.

Stirtz, T. M. 2011. A grammar of Gaahmg: A Nilo-Saharan language of Sudan. Utrecht: LOT.

Stonham, J. 1999. Aspects of Tsishaath Nootka phonetics and phonology. München: Lincom Europa.

Thompson, L. C. and Thompson, M. T. 1992. The Thompson Language. (University of Montana Occasional Papers in Linguistics, 8.) Missoula, Montana: Linguistics Laboratory, University of Montana.

Vajda, E. J. 2004. Ket. (Languages of the World: Materials, 204.) München: Lincom.

Vallejos Yopán, R. 2010. A grammar of Kokama-Kokamilla [dissertation]. [Eugene]: University of Oregon.

van der Leeden, A.C. 1993. Ma'ya: a language study. Phonology. Seri Terbitan LIPI-RUL Jakarta: Lembaga Ilmu Pengetahuan Indonesia and Rijkuniversiteit te Leiden.

Watson, R. L. 1980. A Grammar of Two Pacoh Texts. Ann Arbor: UMI. [dissertation]. [Arlington]: University of Texas at Arlington.

Wegener, C. U. 2008. A grammar of Savosavo: a Papuan language of the Solomon Islands. Wageningen: Ponsen & Looijen.

Watson, R. L. 1980. A Grammar of Two Pacoh Texts. Ann Arbor: UMI. [dissertation]. [Arlington]: University of Texas at Arlington.

Whitehead, C. R. 2004. A reference grammar of Menya, an Angan language of Papua New Guinea. Ann Arbor: UMI.

Wojtylak, K. I. 2017. A Grammar of Murui (Bue): A Witotoan language of Northwest Amazonia. [dissertation]. [Townsville]: James Cook University.

Yumitani, Y. 1998. A phonology and morphology of Jemez Towa. [dissertation]. [Lawrence]: University of Kansas.
