# Supplementary material for: Syllable Complexity and Morphological Synthesis: A Well-Motivated Positive Complexity Correlation Across Subdomains
Source: Front Psychol. 2021 Mar 17;12:638659. doi: 10.3389/fpsyg.2021.638659 (PMC8010299; doi:10.3389/fpsyg.2021.638659)
Supplement: Supplementary file 2 [file Table_2.docx]

**6.2. Appendix B: Language sample, corpus study**

| **Language** | **WALS genus** | **Glottocode** | **Corpus Size  (N word types)** | **Reference** |
| --- | --- | --- | --- | --- |
| Arapaho | Algonquian | [arap1274](http://glottolog.org/resource/languoid/id/arap1274) | 4585 | (Cowell 2020) |
| Ruuli | Bantoid | ruul1235 | 4029 | (Witzlack-Makarevich et al. 2020) |
| Beja | Beja | [beja1238](http://glottolog.org/resource/languoid/id/beja1238) | 6040 | (Vanhove 2020) |
| Mojeño Trinitario | Bolivia-Parana | trin1274 | 1246 | (Rose 2020) |
| Bora | Boran | bora1263 | 10579 | (Seifart 2020) |
| Hoocąk | Core Siouan | hoch1243 | 6306 | (Hartmann 2013) |
| Gurindji Kriol | Creoles and Pidgins | [guri1249](http://glottolog.org/resource/languoid/id/guri1249) | 1693 | (Meakins 2020) |
| Northern Kurdish | Iranian | [nort2641](http://glottolog.org/resource/languoid/id/nort2641) | 1925 | (Haig et al. 2020) |
| Katla | Katla-Tima | katl1237 | 3838 | (Hellwig 2020b) |
| Pnar | Khasian | [pnar1238](http://glottolog.org/resource/languoid/id/pnar1238) | 4665 | (Ring 2020) |
| Sumi | Kuki-Chin | sumi1235 | 1727 | (Teo 2020) |
| Sanzhi Dargwa | Lak-Dargwa | sanz1248 | 1518 | (Forker 2020) |
| Movima | Movima | movi1243 | 1350 | (Haude 2020) |
| Northern Alta | Northern Luzon | nort2875 | 1925 | (Garcia-Laguia 2020) |
| Nafsan | Oceanic | [sout2856](http://glottolog.org/resource/languoid/id/sout2856) | 3404 | (Thieberger 2020) |
| Kamas | Samoyedic | [kama1378](http://glottolog.org/resource/languoid/id/kama1378) | 2581 | (Gusev et al. 2020) |
| Savosavo | Savosavo | savo1255 | 1343 | (Wegener 2020) |
| Evenki | Tungusic | even1259 | 4743 | (Kazakevich & Klyachko 2020) |
| Urum | Turkic | [urum1249](http://glottolog.org/resource/languoid/id/urum1249) | 5848 | (Skopeteas et al. 2020) |
| Goemai | West Chadic | goem1240 | 1682 | (Hellwig 2020a) |
| Kakabe | Western Mande | kaka1265 | 3625 | (Vydrina 2020) |

**References**

Cowell, A. 2020. “Arapaho DoReCo data set,” in Language Documentation Reference Corpus (DoReCo) 1.0, ed. F. Seifart, L. Paschen and M. Stave (Berlin & Lyon: Leibniz-Zentrum Allgemeine Sprachwissenschaft & Laboratoire Dynamique Du Langage UMR5596, CNRS & Université Lyon 2).

Forker, D. 2020. “Sanzhi Dargwa DoReCo data set,” in Language Documentation Reference Corpus (DoReCo) 1.0, ed. F. Seifart, L. Paschen and M. Stave (Berlin & Lyon: Leibniz-Zentrum Allgemeine Sprachwissenschaft & Laboratoire Dynamique Du Langage UMR5596, CNRS & Université Lyon 2).

Garcia-Laguia, A. 2020. “Northern Alta DoReCo data set,” in Language Documentation Reference Corpus (DoReCo) 1.0, ed. F. Seifart, L. Paschen and M. Stave (Berlin & Lyon: Leibniz-Zentrum Allgemeine Sprachwissenschaft & Laboratoire Dynamique Du Langage UMR5596, CNRS & Université Lyon 2).

Gusev, V., Klooster, T., Wagner-Nagy, B., and Arkhipov, A. 2020. “Kamas DoReCo data set,” in Language Documentation Reference Corpus (DoReCo) 1.0, ed. F. Seifart, L. Paschen and M. Stave (Berlin & Lyon: Leibniz-Zentrum Allgemeine Sprachwissenschaft & Laboratoire Dynamique Du Langage UMR5596, CNRS & Université Lyon 2).

Haig, G., Vollmer, M. & Thiele, H. 2020. “Northern Kurdish DoReCo data set,” in Language Documentation Reference Corpus (DoReCo) 1.0, ed. F. Seifart, L. Paschen and M. Stave (Berlin & Lyon: Leibniz-Zentrum Allgemeine Sprachwissenschaft & Laboratoire Dynamique Du Langage UMR5596, CNRS & Université Lyon 2).

Hartmann, I. 2013. Hoocąk Corpus. Leipzig: MPI-EVA.

Haude, K. 2020. “Movima DoReCo data set,” in Language Documentation Reference Corpus (DoReCo) 1.0, ed. F. Seifart, L. Paschen and M. Stave (Berlin & Lyon: Leibniz-Zentrum Allgemeine Sprachwissenschaft & Laboratoire Dynamique Du Langage UMR5596, CNRS & Université Lyon 2).

Hellwig, B. 2020a. “Goemai DoReCo data set,” in Language Documentation Reference Corpus (DoReCo) 1.0, ed. F. Seifart, L. Paschen and M. Stave (Berlin & Lyon: Leibniz-Zentrum Allgemeine Sprachwissenschaft & Laboratoire Dynamique Du Langage UMR5596, CNRS & Université Lyon 2).

Hellwig, B. 2020b. “Katla DoReCo data set,” in Language Documentation Reference Corpus (DoReCo) 1.0, ed. F. Seifart, L. Paschen and M. Stave (Berlin & Lyon: Leibniz-Zentrum Allgemeine Sprachwissenschaft & Laboratoire Dynamique Du Langage UMR5596, CNRS & Université Lyon 2).

Kazakevich, O. and Klyachko, E. 2020. “Evenki DoReCo data set,” in Language Documentation Reference Corpus (DoReCo) 1.0, ed. F. Seifart, L. Paschen and M. Stave (Berlin & Lyon: Leibniz-Zentrum Allgemeine Sprachwissenschaft & Laboratoire Dynamique Du Langage UMR5596, CNRS & Université Lyon 2).

Meakins, F. 2020. “Gurindji Kriol DoReCo data set,” in Language Documentation Reference Corpus (DoReCo) 1.0, ed. F. Seifart, L. Paschen and M. Stave (Berlin & Lyon: Leibniz-Zentrum Allgemeine Sprachwissenschaft & Laboratoire Dynamique Du Langage UMR5596, CNRS & Université Lyon 2).

Ring, H. 2020. “Pnar DoReCo data set,” in Language Documentation Reference Corpus (DoReCo) 1.0, ed. F. Seifart, L. Paschen and M. Stave (Berlin & Lyon: Leibniz-Zentrum Allgemeine Sprachwissenschaft & Laboratoire Dynamique Du Langage UMR5596, CNRS & Université Lyon 2).

Rose, F. 2020. “Mojeño Trinitario DoReCo data set,” in Language Documentation Reference Corpus (DoReCo) 1.0, ed. F. Seifart, L. Paschen and M. Stave (Berlin & Lyon: Leibniz-Zentrum Allgemeine Sprachwissenschaft & Laboratoire Dynamique Du Langage UMR5596, CNRS & Université Lyon 2).

Seifart, F. 2020. “Bora DoReCo data set,” in Language Documentation Reference Corpus (DoReCo) 1.0, ed. F. Seifart, L. Paschen and M. Stave (Berlin & Lyon: Leibniz-Zentrum Allgemeine Sprachwissenschaft & Laboratoire Dynamique Du Langage UMR5596, CNRS & Université Lyon 2).

Skopeteas, S., Moisidi, V., Tsetereli, N., Lorenz, J. and Schröter, S. 2020. “Urum DoReCo data set,” in Language Documentation Reference Corpus (DoReCo) 1.0, ed. F. Seifart, L. Paschen and M. Stave (Berlin & Lyon: Leibniz-Zentrum Allgemeine Sprachwissenschaft & Laboratoire Dynamique Du Langage UMR5596, CNRS & Université Lyon 2).

Teo, A. 2020. “Sumi DoReCo data set,” in Language Documentation Reference Corpus (DoReCo) 1.0, ed. F. Seifart, L. Paschen and M. Stave (Berlin & Lyon: Leibniz-Zentrum Allgemeine Sprachwissenschaft & Laboratoire Dynamique Du Langage UMR5596, CNRS & Université Lyon 2).

Thieberger, N. 2020. “Nafsan DoReCo data set,” in Language Documentation Reference Corpus (DoReCo) 1.0, ed. F. Seifart, L. Paschen and M. Stave (Berlin & Lyon: Leibniz-Zentrum Allgemeine Sprachwissenschaft & Laboratoire Dynamique Du Langage UMR5596, CNRS & Université Lyon 2).

Vanhove, M. 2020. “Beja DoReCo data set, annotated within CorpAfroAs and CORPORAN, reannotated within DORECO,” in Language Documentation Reference Corpus (DoReCo) 1.0, ed. F. Seifart, L. Paschen and M. Stave (Berlin & Lyon: Leibniz-Zentrum Allgemeine Sprachwissenschaft & Laboratoire Dynamique Du Langage UMR5596, CNRS & Université Lyon 2).

Vydrina, A. 2020. “Kakabe DoReCo data set,” in Language Documentation Reference Corpus (DoReCo) 1.0, ed. F. Seifart, L. Paschen and M. Stave (Berlin & Lyon: Leibniz-Zentrum Allgemeine Sprachwissenschaft & Laboratoire Dynamique Du Langage UMR5596, CNRS & Université Lyon 2).

Wegener, C. 2020. “Savosavo DoReCo data set,” in Language Documentation Reference Corpus (DoReCo) 1.0, ed. F. Seifart, L. Paschen and M. Stave (Berlin & Lyon: Leibniz-Zentrum Allgemeine Sprachwissenschaft & Laboratoire Dynamique Du Langage UMR5596, CNRS & Université Lyon 2).

Witzlack-Makarevich, A., Namyalo, S., Kiriggwajjo, A., Molochieva, Z., and Atuhairwe, A. 2020. “Ruuli DoReCo data set,” in Language Documentation Reference Corpus (DoReCo) 1.0, ed. F. Seifart, L. Paschen and M. Stave (Berlin & Lyon: Leibniz-Zentrum Allgemeine Sprachwissenschaft & Laboratoire Dynamique Du Langage UMR5596, CNRS & Université Lyon 2).
